# Establishment of the Inducible Tet-On System for the Activation of the Silent Trichosetin Gene Cluster in *Fusarium fujikuroi*

**DOI:** 10.3390/toxins9040126

**Published:** 2017-04-05

**Authors:** Slavica Janevska, Birgit Arndt, Leonie Baumann, Lisa Helene Apken, Lucas Maciel Mauriz Marques, Hans-Ulrich Humpf, Bettina Tudzynski

**Affiliations:** 1 Institute of Plant Biology and Biotechnology, Westfälische Wilhelms-Universität Münster, Schlossplatz 8, 48143 Münster, Germany; slavica.janevska@uni-muenster.de (S.J.); leoniebaumann@gmx.com (L.B.); l_apke01@uni-muenster.de (L.H.A.); 2 Institute of Food Chemistry, Westfälische Wilhelms-Universität Münster, Corrensstrasse 45, 48149 Münster, Germany; birgit.arndt@uni-muenster.de (B.A.); lucasmauriz@yahoo.com.br (L.M.M.M.); humpf@uni-muenster.de (H.-U.H.)

**Keywords:** Fungi, *Fusarium fujikuroi*, secondary metabolism, PKS-NRPS, biosynthesis, gene regulation

## Abstract

The PKS-NRPS-derived tetramic acid equisetin and its *N*-desmethyl derivative trichosetin exhibit remarkable biological activities against a variety of organisms, including plants and bacteria, e.g., *Staphylococcus aureus*. The equisetin biosynthetic gene cluster was first described in *Fusarium heterosporum*, a species distantly related to the notorious rice pathogen *Fusarium fujikuroi*. Here we present the activation and characterization of a homologous, but silent, gene cluster in *F. fujikuroi*. Bioinformatic analysis revealed that this cluster does not contain the equisetin *N*-methyltransferase gene *eqxD* and consequently, trichosetin was isolated as final product. The adaption of the inducible, tetracycline-dependent Tet-on promoter system from *Aspergillus niger* achieved a controlled overproduction of this toxic metabolite and a functional characterization of each cluster gene in *F. fujikuroi*. Overexpression of one of the two cluster-specific transcription factor (TF) genes, *TF22*, led to an activation of the three biosynthetic cluster genes, including the *PKS-NRPS* key gene. In contrast, overexpression of *TF23*, encoding a second Zn(II)_2_Cys_6_ TF, did not activate adjacent cluster genes. Instead, *TF23* was induced by the final product trichosetin and was required for expression of the transporter-encoding gene *MFS-T*. TF23 and MFS-T likely act in consort and contribute to detoxification of trichosetin and therefore, self-protection of the producing fungus.

## 1. Introduction

*Fusarium fujikuroi* belongs to the *Fusarium* (*Gibberella*) *fujikuroi* species complex (FFC) [1,2], which causes the *bakanae* (“foolish seedling”) disease of rice plants [3]. The chlorosis and hyper-elongation of plant internodes characteristic of this disease occur due to the production of the phytohormone gibberellic acid by *F. fujikuroi* [4,5]. *F. fujikuroi* can also synthesize a variety of other secondary metabolites (SMs) including the polyketide synthase (PKS)-derived bikaverin, fusarubins, fujikurins, fumonisins, and gibepyrones [6,7,8,9,10,11]; the non-ribosomal peptide synthetase (NRPS)-derived apicidin F and beauvericin [12,13]; the PKS-NRPS- or PKS and NRPS-derived fusarins and fusaric acid [14,15,16]; as well as the terpene cyclase-derived (+)-eremophilene, (−)-α-acorenol, (−)-guaia-6,10(14)-diene and (+)-koraiol [17,18]. In addition, *F. fujikuroi* has the genetic potential to synthesize at least 31 more SMs based on the presence of core SM genes and analysis of flanking genes [19].

The tetramic acid (pyrrolidine-2,4-dione) ring system is a characteristic feature of natural products. Naturally occurring tetramic acid derivatives are of great interest because they show a large spectrum of bioactivities, including antibacterial, antiviral, fungicidal, phytotoxic, and cytotoxic effects [20,21]. The tetramic acid equisetin was initially isolated as an antibiotic from cultures of *Fusarium equiseti* that effectively inhibited growth of Gram-positive bacteria such as *Bacillus subtilis* and *Staphylococcus aureus* [22]. Equisetin has also been reported to be toxic to various mono- and dicotyledonous plants [23] and to inhibit the human immunodeficiency virus type 1 integrase [24,25]. Equisetin biosynthesis and the responsible gene cluster have been characterized in *Fusarium heterosporum* and *Fusarium* sp. FN080326 [26,27].

Trichosetin, the penultimate intermediate in equisetin biosynthesis and its *N*-desmethyl derivative, have also been well studied [26]. For example, trichosetin isolated from the dual culture of *Trichoderma harzianum* and *Catharanthus roseus* callus strongly inhibited *B. subtilis* and *S. aureus* growth [28]. Trichosetin has also been found to be active against methicillin-resistant *S. aureus* strains at IC_50_ values of ca. 30 µM, likely by interfering with the enzyme undecaprenyl pyrophosphate synthase required for peptidoglycan synthesis [29,30]. Concerning its possible application as a drug, *rec* assays and micronucleus tests were performed, evaluating its DNA-damaging and chromosome-breaking potential, respectively. Both tests revealed that trichosetin is not mutagenic at the tested concentrations [31]. In an animal model however, trichosetin was found to be toxic to mice, possibly targeting the central nervous system [32]. Furthermore, trichosetin inhibited both root and shoot growth of mono- and dicotyledonous plants (rice, tomato, and chili seedlings) at a concentration of 10 µg/mL, most likely through mitochondrial damage, lipid peroxidation, and subsequent cell membrane damage [33].

The first key enzyme for the biosynthesis of equisetin and its *N*-desmethyl precursor trichosetin has been identified in *F. heterosporum* [26]. The PKS-NRPS together with a *trans*-acting enoyl reductase is suggested to condense an octaketide with a decalin ring system via Diels-Alder cyclization. After fusion of ketide and serine amino acid moieties, trichosetin is most likely released via Dieckmann cyclization, however, mechanistic studies have not been carried out [34]. In a final step, trichosetin is *N*-methylated to obtain equisetin in *F. heterosporum* [26].

In this study, we present the identification, activation, and characterization of the silent trichosetin biosynthetic gene cluster in the rice pathogen *F. fujikuroi*. We show that one of the two cluster-specific transcription factor (TF) genes, *TF22*, encodes the positive cluster regulator, controlling expression of the three biosynthetic genes. In contrast, the second cluster-specific TF gene, *TF23*, is only activated by the final product trichosetin itself, and is probably responsible for the detoxification of trichosetin. The inducible gene expression system Tet-on was adapted from *Aspergillus niger* and established for *F. fujikuroi*, enabling a tunable expression and functional characterization of all cluster genes as well as an inducible production of this toxic metabolite. Additionally, the phytotoxicity and cytotoxicity of trichosetin was evaluated.

## 2. Results

### 2.1. Identification of a Cluster Homologous to the *F. heterosporum* Equisetin Gene Cluster

**Figure 1:**
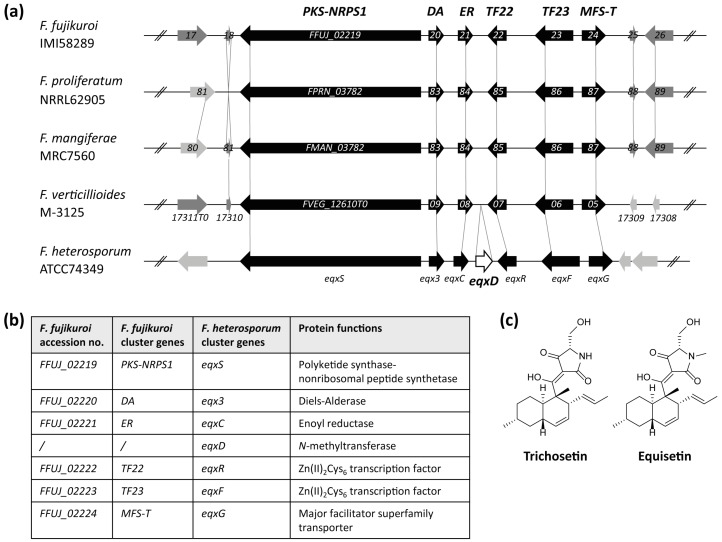
Conservation of the equisetin gene cluster among members of the *F. fujikuroi* species complex (FFC). (**a**) Schematic representation of the equisetin gene cluster found in the FFC (*F. fujikuroi*, *F. proliferatum*, *F. mangiferae,* and *F. verticillioides*) and the respective gene cluster in *F. heterosporum* according to Kakule et al. [26]. Dashed lines indicate homologous genes and homologous cluster genes are represented by black arrows. *F. heterosporum eqxD*, missing within the FFC, is indicated as white arrow; (**b**) Accession numbers (No.), gene names and protein functions of *F. fujikuroi* and *F. heterosporum* cluster genes/proteins; (**c**) Chemical structures of trichosetin and equisetin according to Marfori et al. and Turos et al., respectively [28,36].

Bioinformatic analysis revealed a gene cluster consisting of six genes (*FFUJ_02219*-*FFUJ_02224*) in *F. fujikuroi* that is highly homologous to the *F. heterosporum* equisetin gene cluster [26]. *FFUJ_02219* is homologous to *eqxS* and encodes a putative PKS-NRPS hybrid enzyme, PKS-NRPS1. *FFUJ_02220* is homologous to *eqx3* which has been further studied in *Fusarium* sp. FN080326. The encoded protein does not harbor any domain of known function, but has been shown to be involved in conferring the correct stereochemistry in the Diels-Alder condensation of equisetin, designated Diels-Alderase (DA) [27]. *FFUJ_02221* is homologous to *eqxC* and encodes a putative *trans*-acting enoyl reductase (ER). *FFUJ_02222* and *FFUJ_02223*, homologous to *eqxR* and *eqxF*, encode two putative cluster-specific TFs of the fungus-specific Zn(II)_2_Cys_6_-type and are designated *TF22* and *TF23*. Finally, *FFUJ_02224* is homologous to *eqxG* and encodes a putative transporter of the major facilitator superfamily (MFS), designated *MFS-T* (Figure 1a,b). *F. heterosporum eqxD* is absent from *F. fujikuroi* as well as from other members of the FFC (Figure 1a). The predicted product of *eqxD*, a putative *N*-methyltransferase, catalyzes the last step in equisetin biosynthesis, the *N*-methylation of trichosetin forming equisetin (Figure 1c). The lack of an *eqxD* homolog in the indicated *Fusarium* spp. strongly suggests that trichosetin is the product of this gene cluster. However, neither trichosetin nor equisetin have been reported to be synthesized by any member of the FFC [35].

### 2.2. Activation of the *F. fujikuroi* Trichosetin Gene Cluster

**Figure 2:**
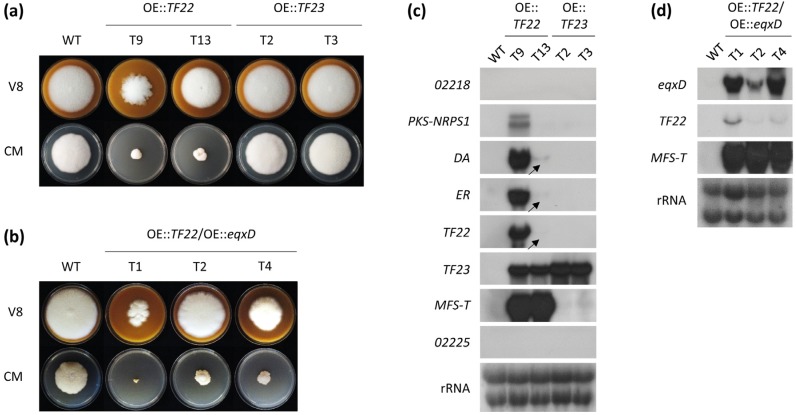
Growth and Northern blot expression analysis of *F. fujikuroi* trichosetin transcription factor (TF) overexpression mutants. (**a**) The wild type (WT) and two independent transformants (T) of OE::*TF22* and OE::*TF23* were grown on V8 and complex medium (CM) for 7 days; (**b**) The WT and three OE::*TF22*/OE::*eqxD* double mutants were grown on V8 and CM for 7 days; (**c**) The WT, OE::*TF22* and OE::*TF23* mutants were grown in liquid culture for 3 days prior to RNA extraction from the harvested mycelium. Blots were probed with DNA corresponding to the six trichosetin cluster genes and the two predicted genes flanking the cluster. Arrows indicate weak hybridization signals; (**d**) The WT and OE::*TF22*/OE::*eqxD* mutants were grown in liquid culture for 3 days prior to RNA extraction. Blots were probed with DNA corresponding to *eqxD*, *TF22,* and *MFS-T*.

Previously, we were not able to detect the expression of the putative trichosetin gene cluster in the *F. fujikuroi* wild type (WT) under any growth condition tested [19]. In order to activate expression of the whole cluster, both of the putative cluster-specific TF genes *TF22* and *TF23* were individually overexpressed (OE) using the constitutive and strong *Aspergillus nidulans PoliC* promoter. While OE::*TF23* mutants did not display any growth defects on V8 complete medium or complex medium (CM), OE::*TF22* transformants exhibited a more (T9) or less (T13) severe growth defect on these media (Figure 2a). This phenotypic difference may be related to the observation that, in the transformant T9, *T22* and all cluster genes *PKS-NRPS1*, *DA*, *ER*, *TF23,* and *MFS-T* were strongly expressed compared to the WT. In contrast, the expression levels were only slightly increased in OE::*TF22* T13 (Figure 2c). The overexpression of *TF23* did not result in an upregulation of any cluster genes (Figure 2c). Therefore, based on these results, TF22 represents the positive cluster-specific TF.

To confirm the chemical identity of the cluster product trichosetin, the culture filtrates of the OE::*TF22* mutants were analyzed via high performance liquid chromatography (HPLC) coupled to high resolution mass spectrometry (HRMS) and compared to the WT. The metabolic profile of the OE::*TF22* mutants contained a peak with a mass consistent with the chemical structure of trichosetin which was not present in the WT (Figure S1). This metabolite was extracted from the supernatant of OE::*TF22* T9 as described under Materials and Methods. Subsequent structure elucidation confirmed the identity of trichosetin. OE::*TF22* T13 produced significantly lower amounts of trichosetin compared to OE::*TF22* T9 (Figure S1), which agrees with the lower level of cluster activation (Figure 2c) and with the more moderate growth defect (Figure 2a) for T13. Such phenotypic differences between two independent transformants have been rarely observed for *F. fujikuroi* and can be most likely attributed to different loci of vector integration (see Materials and Methods) and subsequent differences in toxin accumulation. The toxicity of trichosetin on the producing fungus is further evaluated below.

**Figure 3:**
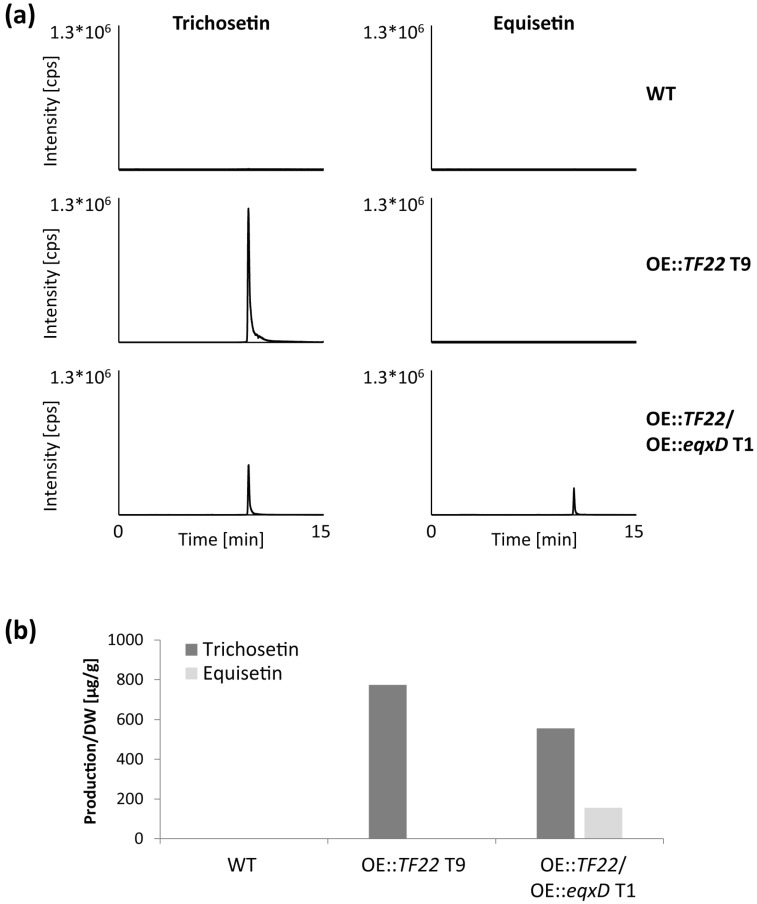
HPLC-tandem mass spectrometry (HPLC-MS/MS) analysis of trichosetin- and equisetin-producing *F. fujikuroi* transcription factor (TF) mutants. (**a**) Qualitative analysis of trichosetin (retention time 9.56 min) and equisetin (retention time 10.49 min) production of the wild type (WT) and two transformants (T) of OE::*TF22* and OE::*TF22*/OE::*eqxD* upon growth in liquid culture for 7 days. cps, counts per second; (**b**) Semi-quantitative analysis of metabolite production related to the dry weight (DW) of the strains. The cultivation was done in duplicate.

To provide additional evidence for the homology of the *F. fujikuroi* and *F. heterosporum* gene clusters, the *N*-methyltransferase-encoding gene *eqxD* was heterologously expressed under the control of the strong *F. fujikuroi* glutamine synthetase promoter *PglnA* in the OE::*TF22* T9 background (Figure 2d). Growth studies of three independent *eqxD* overexpressing mutants (OE::*TF22*/OE::*eqxD*) indicated that they shared significant phenotypic differences (Figure 2b). HPLC-tandem mass spectrometry (HPLC-MS/MS) analysis verified the successful accumulation of the *N*-methylated product equisetin in liquid cultures, although the conversion was incomplete and significant amounts of trichosetin were still detected in the double mutant (Figure 3a). For example, OE::*TF22* T9 produced around 770 µg trichosetin and no equisetin, while OE::*TF22*/OE::*eqxD* T1 produced around 560 µg trichosetin and 160 µg equisetin per g fungal dry weight (Figure 3b). Also in this experiment, the accumulation of the toxic metabolite(s) trichosetin (and equisetin) is most likely the reason for the observed growth defect (Figure 2b).

### 2.3. Inducible Overexpression of *TF22*

Because the constitutive overexpression of *TF22* resulted in an inconsistent phenotype, possibly due to a growth inhibiting effect of trichosetin on the producing strains (Figure 2a), we developed an inducible gene expression system for *F. fujikuroi* that would allow a controlled production of trichosetin and thus enable the functional characterization of each cluster gene. The bacterial-fungal hybrid promoter system Tet-on previously established for *A. niger* [37] was chosen and adapted for *F. fujikuroi* by placing TET expression under the control of the constitutive strong *PoliC* promoter (instead of *PgpdA* for *A. niger*). The first part of this TET construct encodes the tetracycline-dependent transactivator rtTA2^S^-M2, while the second part harbors the rtTA2^S^-M2-dependent promoter that is fused to the gene of interest, in this case *TF22*. The transactivator rtTA2^S^-M2 was originally taken from *Escherichia coli*, but it has been optimized for eukaryotic systems. When rtTA2^S^-M2 binds the inducer tetracycline, or its structural analog doxycycline (Dox), it initiates gene expression through binding to the operator sequence [37]. For this work, we chose to target the resulting vector pTET::*TF22* to the locus of the highly and constitutively expressed DNA damage repair gene *DDR48* to achieve a consistent and reproducible level of induction in various genetic backgrounds. Furthermore, *DDR48* deletion showed that the encoded protein is not needed for normal growth, development or SM biosynthesis (data not shown).

The WT and single deletion mutants of the five cluster genes (Δ*PKS-NRPS1*, Δ*DA*, Δ*ER*, Δ*TF23*, Δ*MFS-T*) were transformed with pTET::*TF22*. For the analysis of cluster gene expression via quantitative real-time PCR (qRT-PCR), the WT, TET::*TF22* and the five double mutants were grown on solid CM supplemented with 0, 10, or 50 µg/mL Dox. The Dox concentration was not chosen to be any higher, because the addition of 50 µg/mL Dox to solid cultures already reduced growth of the WT to ca. 70% (Figure S2).

**Figure 4:**
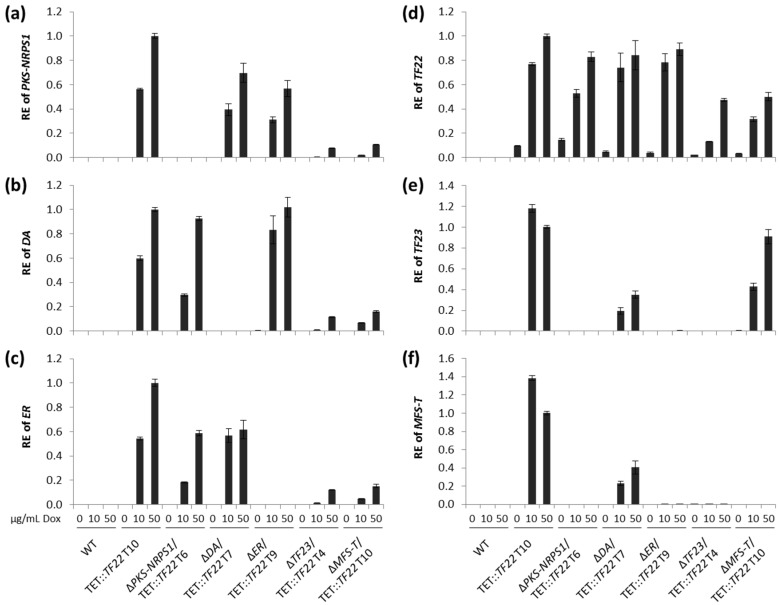
Real-time expression analysis of TET::*TF22* double mutants. The wild type (WT), TET::*TF22* and indicated double mutants were grown on solid CM for 3 days. The medium was supplemented with 0, 10 or 50 µg/mL doxycycline (Dox) for induction of transcription factor (TF) gene expression. Total RNA was isolated from the harvested mycelium, transcribed into cDNA and the relative expression (RE) was analyzed using the ΔΔCt method for (**a**) *PKS-NRPS1*; (**b**) *DA*; (**c**) *ER*; (**d**) *TF22*; (**e**) *TF23* and (**f**) *MFS-T*. Error bars (±standard deviation) originate from a technical replicate and expression of TET::*TF22*, 50 µg/mL Dox was arbitrarily set to 1. T, transformant.

For all strains containing the TET::*TF22* vector, we observed a strong activation of *TF22* expression in a dose-dependent manner (Figure 4d; Figure S3). Unfortunately, the system was found to be “leaky”, as a basal expression of *TF22* was observed in the absence of inducer. This could be due to the placement of the TET construct at the *DDR48* locus which, as noted previously, is generally highly expressed [38]. However, this basal level of *TF22* was insufficient to activate cluster gene expression (Figure 4a–c,e,f). Therefore, the system was still considered to be suitable and appropriate for the tunable induction of trichosetin cluster genes.

**Figure 5:**
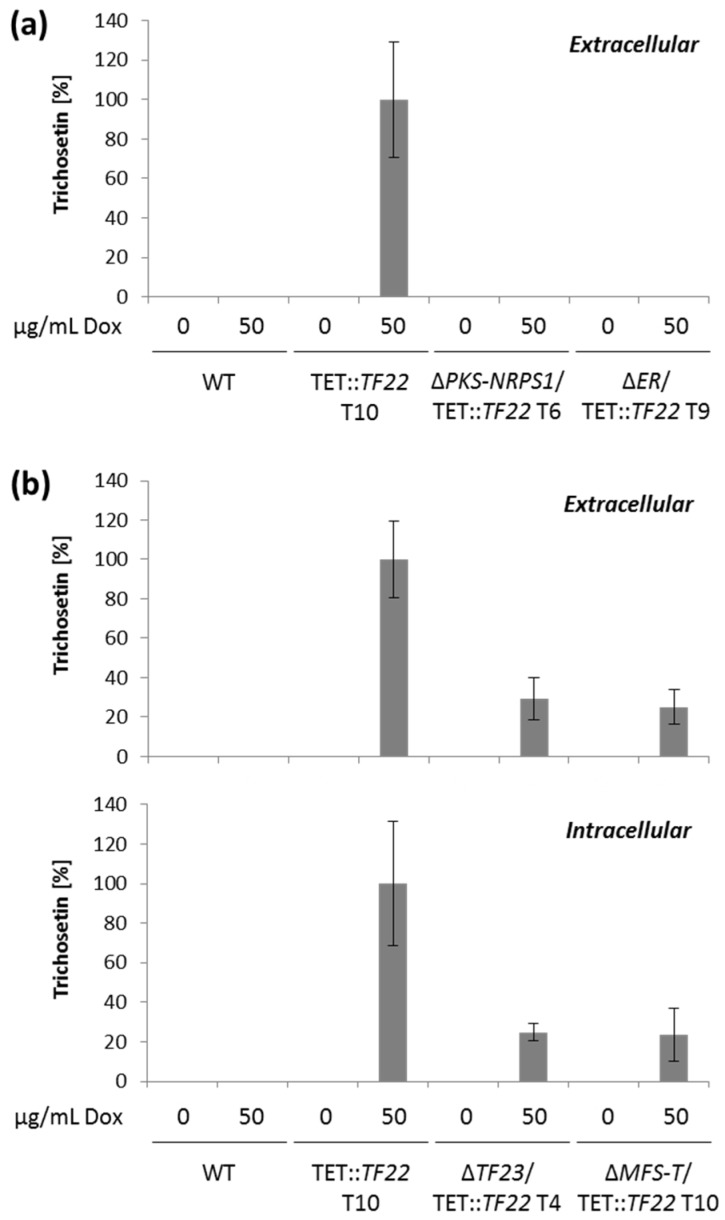
HPLC-MS/MS analysis of TET::*TF22* double mutants. (**a**) The wild type (WT), TET::*TF22* as well as Δ*PKS-NRPS1*/TET::*TF22* and Δ*ER*/TET::*TF22* double mutants were grown in liquid culture for 2 days, then transcription factor (TF) gene expression was induced with 0 or 50 µg/mL doxycycline (Dox) for an additional 5 days. The supernatant was analyzed without further processing; (**b**) The WT, TET::*TF22* as well as Δ*TF23*/TET::*TF22* and Δ*MFS-T*/TET::*TF22* double mutants were grown in liquid culture for 2 days, then TF gene expression was induced with 0 or 50 µg/mL Dox for an additional 5 days. The supernatant was analyzed without further processing for extracellular trichosetin contents, while metabolite extraction from washed mycelium was performed prior to analysis of intracellular trichosetin levels. Both cultivations were done in triplicates. Trichosetin production was related to the dry weight of the samples and the production level of TET::*TF22*, 50 µg/mL Dox was set to 100%. T, transformant.

To determine trichosetin levels via HPLC-MS/MS, the strains were grown in liquid culture. The inducing agent (50 µg/mL Dox) was added after 48 h when the initial growth phase was complete, whereupon the cultivation was continued for five days. Trichosetin was successfully produced by TET::*TF22* (WT background) when induced with Dox, whereas expression of *TF22* in the Δ*PKS-NRPS1* and Δ*ER* deletion backgrounds completely abolished trichosetin production (Figure 5a), thus providing evidence that the encoded enzymes are essential for trichosetin biosynthesis. Concerning cluster gene expression, all cluster genes (the biosynthetic genes *PKS-NRPS1*, *DA,* and *ER* as well as *TF23* and *MFS-T*) were expressed in TET::*TF22* when induced with Dox. In contrast, Δ*PKS-NRPS1*/TET::*TF22* and Δ*ER*/TET::*TF22* only expressed the two remaining biosynthetic genes, respectively, but not *TF23* or *MFS-T* (Figure 4a–c,e,f). These results suggest that TF22 directly activates expression of only the three biosynthetic genes *PKS-NRPS1*, *DA* and *ER*, while *TF23* and *MFS-T* are induced by the final product trichosetin and not by TF22.

Deletion of *DA* resulted in the production of reduced, though significant, amounts of trichosetin (Figure S4) and the expression of *TF23* and *MFS-T* in Δ*DA*/TET::*TF22* mutants when induced with Dox (Figure 4e,f). However, two additional peaks were identified in Δ*DA*/TET::*TF22* mutants with precisely the same mass as trichosetin (Figure S4) and with similar fragmentation patterns (Table S1) that most likely represent stereoisomers of trichosetin.

Dox-mediated induction of *TF22* in Δ*TF23* and Δ*MFS-T* deletion backgrounds resulted in reduced accumulation of only ca. 25% trichosetin compared to the WT background (Figure 5b). This reduced production is consistent with a concomitant reduction in expression of the biosynthetic genes *PKS-NRPS1*, *DA,* and *ER* (Figure 4a–c). Interestingly, the accumulation of trichosetin in Δ*TF23*/TET::*TF22* appeared to be unable to induce expression of *MFS-T*, suggesting that TF23 is necessary for *MFS-T* induction (Figure 4f). Analysis of extra- and intracellular trichosetin levels showed that trichosetin was still secreted upon deletion of the transporter-encoding gene *MFS-T* (Figure 5b).

**Figure 6:**
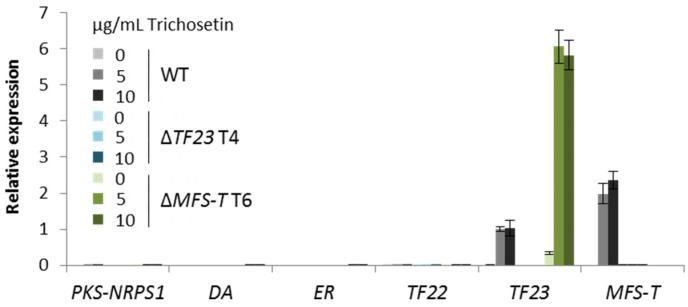
Real time expression analysis of cluster genes after trichosetin feeding in liquid culture. The wild type (WT; gray), Δ*TF23* (blue), and Δ*MFS-T* (green) transformants (T) were grown for 2 days, then 0, 5 or 10 µg/mL trichosetin was added for an additional 2 h. Total RNA was isolated from the harvested mycelium, transcribed into cDNA and the relative expression was analyzed using the ΔΔCt method for the six cluster genes. Error bars (±standard deviation) originate from a technical replicate and *TF23* expression of the WT, 5 µg/mL trichosetin was arbitrarily set to 1.

In order to further analyze the induction of *TF23* and *MFS-T* by trichosetin, we exposed 0, 5 or 10 µg/mL of the purified trichosetin to liquid cultures of the WT, Δ*TF23* and Δ*MFS-T* (Figure 6). Indeed, only expression of *TF23* and *MFS-T*, but not of *TF22* or the biosynthetic genes, was induced in the WT. As already indicated above, the expression of *MFS-T* could not be induced in the Δ*TF23* deletion background. The observation that *TF23* expression was 6-fold upregulated in Δ*MFS-T* compared to the WT (Figure 6) suggests that TF23 might affect the expression of other genes in addition to *MFS-T*.

### 2.4. Trichosetin Is Toxic to the Producing Fungus

**Figure 7:**
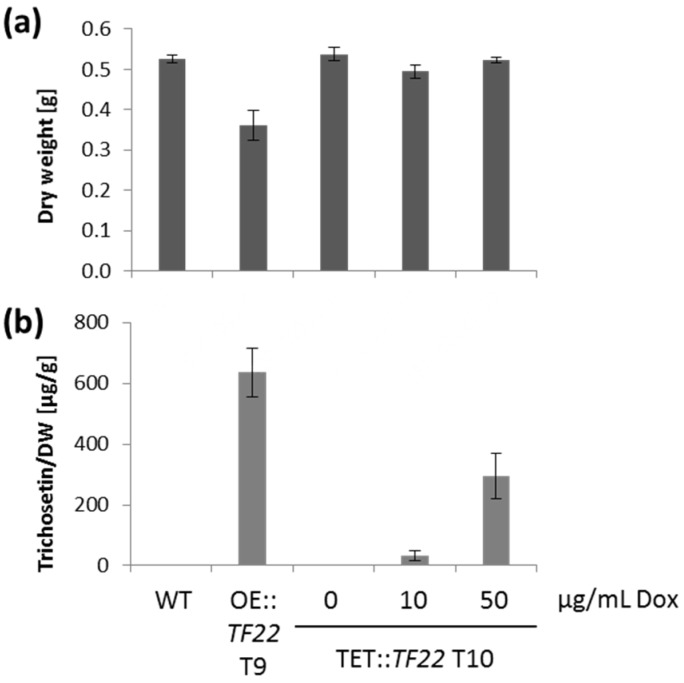
Comparison of constitutive and inducible transcription factor (TF) overexpression. The wild type (WT), OE::*TF22* and TET::*TF22* transformants (T) were grown in liquid culture for 7 days. In the case of TET::*TF22*, the strain was grown for 2 days, then *TF22* gene expression was induced with 0, 10, or 50 µg/mL doxycycline (Dox) for an additional 5 days. The cultivation was done in triplicate. (**a**) The dry weight (DW) of the cultures was determined upon harvest and freeze drying of the mycelium; (**b**) Semi-quantitative HPLC-MS/MS analysis of trichosetin production related to the DW of the strains.

Our observation that the constitutive overexpression of *TF22* resulted in a rather severe phenotype (Figure 2a) strongly suggested a toxic effect of trichosetin on the producing fungus. In order to study this further, we compared the constitutive overexpression mutant OE::*TF22* with TET::*TF22* in liquid cultures induced with 0, 10 or 50 µg/mL Dox on the second day of cultivation. Interestingly, the addition of 50 µg/mL Dox to the liquid cultures did not impact fungal growth which stands in marked contrast to the significant reduction in growth observed for Dox solid cultures (Figure S2). Accumulation of trichosetin did not inhibit growth of TET::*TF22* after induction, whereas OE::*TF22* showed a significantly reduced fungal biomass in liquid cultures (Figure 7a). In fact, TET::*TF22* induced with 50 µg/mL Dox reached only 50% of the trichosetin level produced by OE::*TF22*. Thus, TET::*TF22* produced ~290 µg trichosetin per g fungal dry weight, whereas OE::*TF22* T9 produced ~640 µg/mL in the same culture condition (Figure 7b). The reduced production of trichosetin by TET::*TF22* may explain why TET::*TF22* was not reduced in growth in liquid cultures.

**Figure 8:**
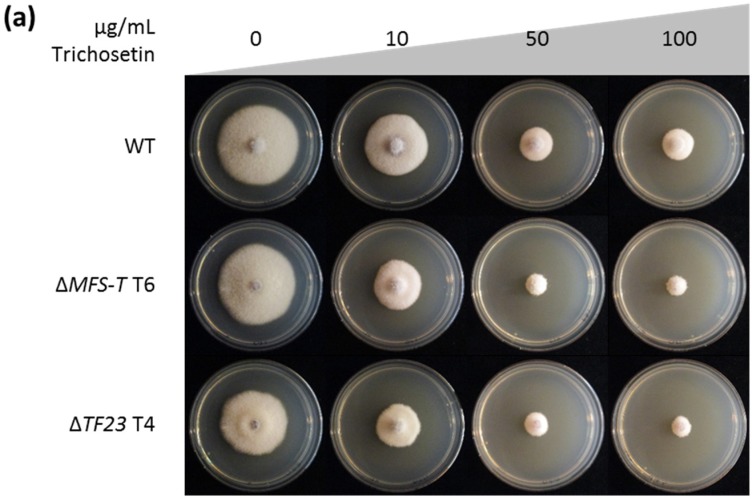

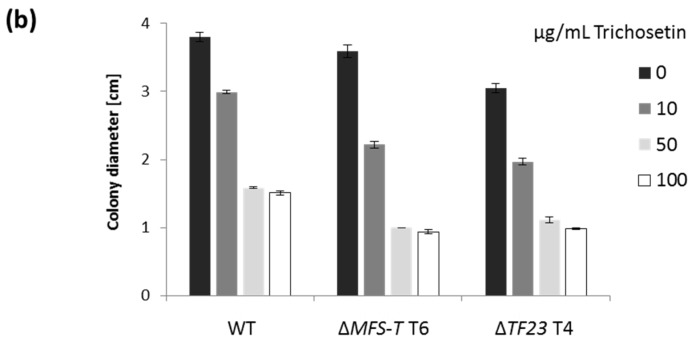
Trichosetin plate assay. (**a**) The wild type (WT), Δ*MFS-T* and Δ*TF23* transformants (T) were grown on solid CM supplemented with 0, 10, 50, or 100 µg/mL trichosetin for 4 days; (**b**) The cultivation was done in triplicate and average colony diameters are shown.

To provide further proof of the toxic effect of trichosetin on *F. fujikuroi* and to analyze the role of TF23 and MFS-T in the detoxification of this metabolite, we inoculated agar plates containing different concentrations of the purified trichosetin (10–100 µg/mL) with the WT and *TF23* and *MFS-T* deletion mutants. The presence of 10 or 100 µg/mL trichosetin significantly reduced growth of the WT to ca. 80% or 40%, respectively. Indeed, the effect on the two deletion mutants Δ*TF23* and Δ*MFS-T* was more severe: 10 or 100 µg/mL trichosetin reduced their colony diameter to ca. 60% or 25%, respectively (Figure 8; Figure S5). In summary, the toxicity of trichosetin to the *F. fujikuroi* WT and greater toxicity to the *TF23* and *MFS-T* deletion mutants support our hypothesis that these two genes are likely involved in its detoxification.

### 2.5. Pathogenicity of Trichosetin- and Equisetin-Producing Mutants on Rice

**Figure 9:**
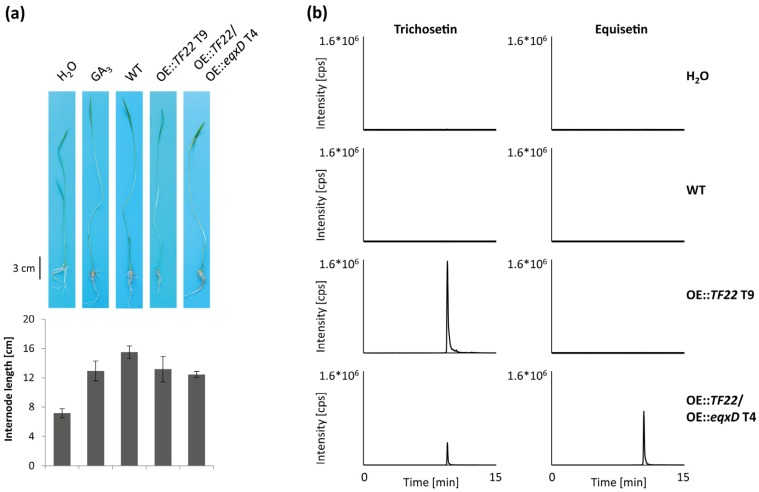
Pathogenicity on rice of trichosetin- and equisetin-producing *F. fujikuroi* transcription factor (TF) mutants. (**a**) Germinated rice seedlings were infected with H_2_O (negative control), 100 ppm gibberellic acid GA_3_ (positive control), the wild type (WT) as well as two transformants (T) of OE::*TF22* and OE::*TF22*/OE::*eqxD*. Three rice plants per sample were analyzed after 7 days-post-infection; (**b**) Qualitative analysis of trichosetin (retention time 9.56 min) and equisetin (retention time 10.49 min) production via HPLC-MS/MS. Seven rice plants per sample were combined, freeze dried and extracted prior to metabolite analysis. cps, counts per second.

As trichosetin and equisetin have both been described as phytotoxic [23,33], we tested the pathogenicity of trichosetin- and equisetin-producing *F. fujikuroi* mutants. Germinated rice seedlings were infected with the WT as well as with the OE::*TF22* and OE::*TF22*/OE::*eqxD* mutants. However, all three strains caused comparable and typical *bakanae* symptoms, i.e. chlorotic hyper-elongated internodes, and comparable levels of necrosis (Figure 9a). HPLC-MS/MS of extracted rice plants verified that the two overexpression mutants produced trichosetin and equisetin, not only in vitro, but also in planta (Figure 9b).

We then examined if trichosetin might affect germinating rice seedlings rather than growth after seed germination. In this experiment, we incubated rice seedlings with mycelium of the WT or OE::*TF22* before germination. We found that the WT reduced shoot growth to 76% and root growth to 44% of the H_2_O control, while incubation with the trichosetin producer OE::*TF22* had a more severe effect, enabling shoot growth in only 63% and root growth in only 24% of the samples (Figure S6).

### 2.6. Cytotoxicity of Trichosetin and Equisetin

Finally, to evaluate the cytotoxic properties of trichosetin and equisetin, the chemicals were applied to the liver cancer cell line Hep G2 in a cell proliferation and cytotoxicity assay (cell counting kit-8, CCK-8; Figure S7). With this test, cellular dehydrogenase activity was measured to determine viability. Both substances had an IC_50_ value below concentrations of 50 µM. Equisetin showed a lower IC_50_ value of 20.5 ± 0.9 µM in comparison to trichosetin with an IC_50_ value of 38.7 ± 4.4 µM (Figure S7).

## 3. Discussion

In the present work, we activated a heretofore silent gene cluster in the rice pathogen *F. fujikuroi* by two different approaches and found that it is responsible for the synthesis of trichosetin, the penultimate intermediate in the synthesis of equisetin. The identification of the equisetin gene cluster and the functional characterization of structural genes, including the PKS-NRPS, were described some time ago for distantly related *Fusarium* species [26,27]. Our work, for the first time, defines the specialized roles of the two TFs encoded by genes in the cluster. Next to the fusarins [14], trichosetin represents the second *F. fujikuroi* SM that is produced by a hybrid PKS-NRPS key enzyme. In contrast, fusaric acid biosynthesis involves two separate key enzymes [16].

### 3.1. Conservation of the Equisetin/Trichosetin Gene Cluster among the Genus *Fusarium*

Equisetin production has been reported in *F. equiseti*, *F. heterosporum*, *Fusarium* sp. FN080326 as well as *Fusarium pallidoroseum* [22,23,26] and the respective gene clusters are highly conserved in the first three strains [27,35]. Our bioinformatic analysis revealed that most of the equisetin gene cluster is also present and highly conserved among members of the FFC. In the FFC, the cluster is missing the gene *eqxD* that is responsible for the last step in equisetin synthesis, the *N*-methylation of trichosetin. Here we report that the actual final product of the predicted gene cluster in *F. fujikuroi* is trichosetin and that expression of *eqxD* from *F. heterosporum* in *F. fujikuroi* led to the production of equisetin. Based on the conserved nature of the gene cluster, trichosetin can be expected as final product for other members of the FFC. The reported synthesis of trichosetin by *Fusarium oxysporum* [29,35], a species more closely related to members of the FFC than *F. heterosporum*, suggests that the loss of *eqxD* may represent an early evolutionary event.

### 3.2. Biosynthesis of Trichosetin Is Regulated by TF22

Activation of the trichosetin gene cluster was achieved through constitutive as well as inducible overexpression of the positive cluster regulator gene *TF22*. Overexpression of *TF22* induced expression of the three biosynthetic genes *PKS-NRPS1*, *DA,* and *ER.* Furthermore, we showed that PKS-NRPS1 and ER are essential for trichosetin biosynthesis because trichosetin production was abolished in the respective deletion mutants. Deletion of the *PKS-NRPS1* and *ER* homologs in *F. heterosporum* and *Fusarium* sp. FN080326 also resulted in a loss of equisetin production [26,27]. All hybrid PKS-NRPS enzymes have the same evolutionary origin, lacking the PKS-specific enoyl reductase module. Although most other fungal PKS-NRPS clusters include genes that encode a *trans*-acting enoyl reductase enzyme (e.g., the tenellin and aspyridone clusters [39]), some clusters do not (e.g., the fusarin cluster [14]).

In contrast to *PKS-NRPS1* and *ER*, deletion of *DA* resulted in the accumulation of significant amounts of trichosetin as well as two additional peaks that most likely represent stereoisomers of trichosetin. In *Fusarium* sp. FN080326, deletion of the *DA* homolog *fsa2* also yielded a novel peak which was identified as the (3*S*,6*R*)-diastereomer of equisetin [27]. The authors suggested that the protein encoded by *fsa2* assists in the *endo*-selective Diels-Alder cycloaddition to form the equisetin decalin ring. Indeed, the decalin ring of tetramic acids can occur in four different stereochemical forms [27] which could explain the presence of two novel trichosetin-like peaks upon deletion of *F. fujikuroi DA*.

**Figure 10:**
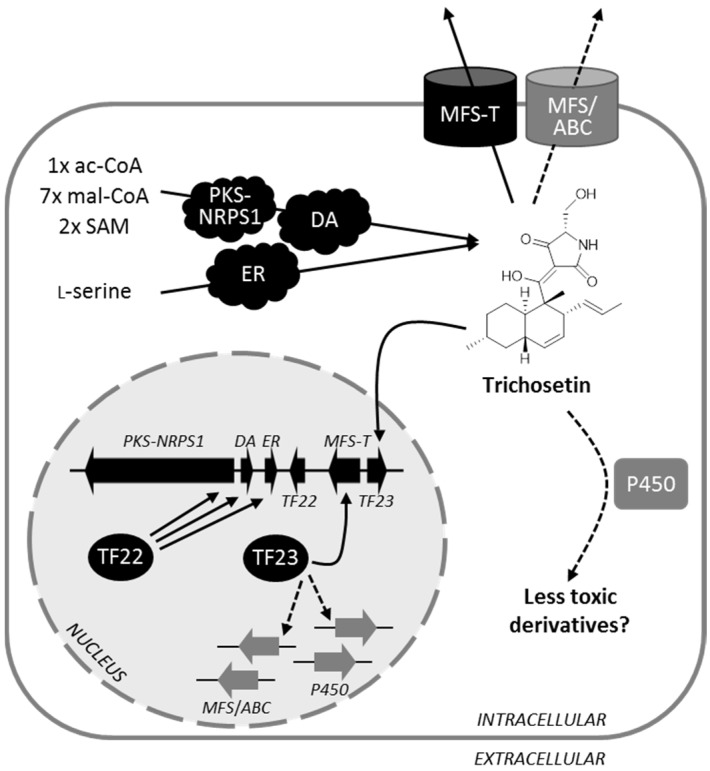
Schematic representation of trichosetin biosynthesis and detoxification, regulated by two separate cluster-specific transcription factors (TFs) in *F. fujikuroi*. Overexpression of the positive cluster regulator gene *TF22* was shown to activate expression of the three biosynthetic genes *PKS-NRPS1*, *DA* (Diels-Alderase) and *ER* (enoyl reductase). The trichosetin carbon skeleton is most likely formed from one acetyl-CoA (ac-CoA), seven malonyl-CoA (mal-CoA), two *S*-adenosyl-l-methionine (SAM), and l-serine [40]. Accumulation of the toxic metabolite trichosetin, in turn, induces expression of the second TF-encoding gene *TF23* which is necessary for the expression of *MFS-T*, encoding a transporter of the major facilitator superfamily. TF23 may also affect the expression of additional genes that may contribute to protection against trichosetin by chemical modification or transport (dashed lines).

Therefore, *F. fujikuroi* PKS-NRPS1, ER, and DA work together to synthesize the tetramic acid trichosetin. As underlined by the feeding of isotopically labeled precursors [40], these enzymes use one acetyl-CoA, seven malonyl-CoA, two *S*-adenosyl-l-methionine, as well as the proteinogenic amino acid l-serine to condense this SM (Figure 10).

### 3.3. Detoxification of Trichosetin Is Likely Regulated by TF23

The role of the *F. fujikuroi* TF23 homologs EqxF and Fsa5 in *F. heterosporum* and *Fusarium* sp. FN080326, respectively, is still unresolved [26,27]. Unlike *TF22*, overexpression of *F. fujikuroi TF23* did not result in activation of any cluster gene. *TF23* expression was not induced in Δ*PKS-NRPS1* and Δ*ER* deletion backgrounds, but was activated by trichosetin feeding, suggesting that expression of *TF23* is specifically induced in the presence of trichosetin independently of the positive cluster regulator TF22. Upon deletion of *TF23*, expression of the transporter-encoding gene *MFS-T* could no longer be induced in the presence of trichosetin. Thus, although TF23 is essential for *MFS-T* expression, overexpression of *TF23* alone is insufficient to induce *MFS-T* expression. Further research is needed to identify potential interaction partners of TF23 that are required for its full activity and that may also be induced by trichosetin.

The presence of trichosetin in solid media significantly impaired WT growth and indicated that trichosetin is toxic to the producing fungus. The observation that both Δ*TF23* and Δ*MFS-T* deletion mutants were more severely impaired compared to the WT suggested that the activation of *MFS-T* by TF23 may likely contribute to detoxification by transporting trichosetin from the fungus. However, deletion of *MFS-T* still facilitated the secretion of trichosetin across the membrane, because it was shown that the SM accumulated both intra- and extracellularly in Δ*MFS-T*/TET::*TF22* mutants. We assume that there are further cluster-independent transporters of the MFS or ATP-binding cassette (ABC) transporter-type that are activated in the absence of MFS-T. As *TF23* expression was more strongly upregulated in the Δ*MFS-T* mutants, it is possible that TF23 may affect the regulation of other targets besides *MFS-T*, such as these cluster-independent MFS or ABC transporters (Figure 10). However, further research is required to address this question.

Interestingly, deletion of *TF23* or *MFS-T* resulted in the downregulation of the three biosynthetic genes and in a reduced accumulation of trichosetin, suggesting a negative feedback loop to control trichosetin biosynthesis when the two detoxifying genes are missing. Similarly, deletion of the *F. fujikuroi TF23* and *MFS-T* homologs *fsa5* and *fsa6* in *Fusarium* sp. FN080326 resulted in a downregulation of equisetin biosynthesis [27].

Fungal SM gene clusters harboring two TF genes are rare. We recently characterized the *F. fujikuroi* fusaric acid gene cluster that also encodes two cluster-specific TFs of the fungus-specific Zn(II)_2_Cys_6_-type, Fub10 and Fub12, as well as a MFS transporter, Fub11 [16]. The trichosetin and fusaric acid gene clusters have in common that TF22/Fub10 only activates transcription of biosynthetic genes, whereas TF23/Fub12 and MFS-T/Fub11 are activated by the final product itself via a yet unknown mechanism. One key difference is that Fub12 does not regulate the transporter-encoding gene in the same manner as TF23, but Fub12 most likely controls the expression of cluster-independent P450 monooxygenase genes that detoxify fusaric acid into less toxic derivatives [16]. Intriguingly, HPLC-HRMS analysis identified a peak in the OE::*TF22* mutant but not in the WT with a mass (*m/z* [M + H]^+^ 376.2118) that could correspond to a trichosetin derivative with a hydroxy or keto group (Figure S8). A hydroxylated *N*-desmethyl derivative of equisetin with this exact mass, designated *N*-demethylophiosetin, had been isolated from co-cultures of *F. pallidoroseum* with the bacterium *Saccharopolyspora erythraea*. In a cytotoxicity assay, *N*-demethylophiosetin was essentially inactive in sharp contrast to equisetin which was active [41]. Taken together, this preliminary data suggests that TF23 may target other genes than *MFS-T*, perhaps encoding one or several P450 monooxygenases, which serve to detoxify trichosetin (Figure 10). It is noteworthy that both trichosetin and fusaric acid are toxic to the producing fungus [16], having a complex network of regulation and detoxification mediated by two cluster-specific TFs.

### 3.4. Phytotoxicity and Cytotoxicity of Trichosetin

Our pathogenicity tests suggest that the in planta trichosetin production by OE::*TF22* did not appear to have a significant phytotoxic effect. It should be noted that a minimal phytotoxic effect could have been masked by disease symptoms generated by the concomitant production of gibberellic acid (e.g., *bakanae* disease). In contrast, we did find that infecting rice seeds with the trichosetin producer OE::*TF22* prior to germination severely affected especially root growth, but also shoot growth. The application of purified trichosetin at a concentration of 10 µg/mL inhibited the germination of several mono- and dicotyledonous plants, including rice [33]. Also in the latter case, especially root growth was negatively affected in the presence of trichosetin [33].

Trichosetin was also found to be cytotoxic to the liver cancer cell line Hep G2. Interestingly, equisetin, the *N*-methyl derivative of trichosetin, was slightly more toxic. However, further analyses are required, possibly including animal models, to evaluate the cytotoxicity and the underlying mode of action of these two antibiotic compounds.

In conclusion, by activating the silent trichosetin gene cluster in *F. fujikuroi*, we were able to provide a comprehensive overview on the biosynthesis and regulation of this toxic SM. We demonstrate that one of the two cluster-specific TFs, TF22, controls biosynthesis of trichosetin whereas TF23 appears to regulate self-protection from the metabolite.

## 4. Materials and Methods

### 4.1. Fungal Strains, Media, and Growth Conditions

The WT strain *F. fujikuroi* IMI58289 (Commonwealth Mycological Institute, Kew, UK) was used as parental strain for the generation of deletion and overexpression mutants. Furthermore, *F. heterosporum* ATCC 74349 [26] was the source material for the *N*-methyltransferase-encoding gene *eqxD* that was expressed in *F. fujikuroi* IMI58289.

Hyphal growth was assessed on solid V8 (20%, *v*/*v*, vegetable juice; Campbell Food, Puurs, Belgium) or CM [42] media. Between 0 and 100 µg/mL purified trichosetin (dissolved in 100% methanol, MeOH) was added to CM agar after sterilization. Each trichosetin dilution was adjusted to the same volume with MeOH and plates containing only MeOH did not inhibit fungal growth (0 µg/mL trichosetin). The strains were incubated at 28 °C in the dark for 7 and 4 days, respectively. Prior to DNA isolation, strains were grown on CM agar covered with a layer of Cellophane for 3 days under indicated conditions. Also, for RNA isolation from TET::*TF22* mutants, 0–50 µg/mL Dox hyclate (in H_2_O; Sigma-Aldrich, Steinheim, Germany) was added to CM Cellophane plates.

Liquid cultures were incubated for 3 days in 300 mL-Erlenmeyer flasks with 100 mL Darken medium [43], shaken at 180 rpm and 28 °C in the dark. Then 0.5% (*v*/*v*) of this pre-culture served as an inoculum for the main culture. For the analysis of OE::*TF22*, OE::*TF23,* and OE::*TF22*/OE::*eqxD* mutants, the main culture consisted of 100 mL synthetic ICI medium (Imperial Chemical Industries Ltd., London, UK) [44] supplemented with 60 mM glutamine as sole nitrogen source in 300 mL-Erlenmeyer flasks. The strains were grown for 3 or 7 days for gene expression or product formation, respectively. For the analysis of TET::*TF22* mutants, the main culture consisted of 30 mL ICI medium (60 mM glutamine) in 100 mL-Erlenmeyer flasks, while 0–50 µg/mL Dox hyclate (in H_2_O; Sigma-Aldrich, Steinheim, Germany) was added on the second day, and cultivation continued for 5 days. Trichosetin feeding in liquid culture was achieved through the addition of 0–10 µg/mL trichosetin to 30 mL ICI cultures on the second day of cultivation for an additional 2 h. For protoplast transformation of *F. fujikuroi*, 0.5% (*v*/*v*) of the pre-culture was transferred to 100 mL ICI medium with 10 g/L fructose instead of glucose as well as 0.5 g/L (NH_4_)_2_SO_4_ as nitrogen source and shaken for no longer than 16 h.

### 4.2. Plasmid Constructions

All deletion and overexpression vectors were cloned by yeast recombinational cloning [45,46]. For targeted gene deletion, ca. 1 kb large upstream (5′) and downstream (3′) regions were amplified with primer pairs 5F/5R and 3F/3R, respectively (Table S2). The hygromycin resistance cassette *hphR*, containing the hygromycin B phosphotransferase gene *hph* under the control of the *PtrpC* promoter from *A. nidulans*, was amplified with hph_F/hph_R (Table S2) from the template pCSN44 [47]. *Saccharomyces cerevisiae* FY834 [48] was transformed with the obtained fragments as well as with the *Eco*RI/*Xho*I restricted shuttle vector pRS426 [49], resulting in the deletion vectors pΔ*PKS-NRPS1*, pΔ*DA*, pΔ*ER*, pΔ*TF23,* and pΔ*MFS-T*.

The constitutive overexpression of *TF22* and *TF23* was achieved through fusion with the *PoliC* promoter from *A. nidulans*. The full-length gene *TF22* including ca. 200 bp of the native terminator sequence was amplified with TF22_OE_F/TF22_OE_R (Table S3), while the first 1.2 kb of *TF23* was amplified with TF23_OE_F/TF23_OE_R (Table S3). *S. cerevisiae* was transformed with one of these fragments as well as with the *Nco*I/*Sac*II restricted plasmid pNDH-OGG [46], yielding overexpression vectors pOE::*TF22* (Figure S9a) and pOE::*TF23* (Figure S9b). For constitutive overexpression of *eqxD* from *F. heterosporum* in *F. fujikuroi*, the full-length gene *eqxD* [26] was amplified with primer pair eqxD_OE_F/eqxD_OE_R (Table S3) and fused with the *PglnA* promoter from *F. fujikuroi*. The *Nco*I/*Not*I restricted plasmid pNAN-GGT (generated from pNAN-AGT as described by Studt et al. [16]) served as plasmid backbone, yielding pOE::*eqxD* (Figure S9c).

For the inducible overexpression of *TF22*, the vector pTET was generated. In two overlapping fragments (for mutation of the *Not*I restriction site), the relevant parts (*rtTA2^S^-M2*, *TcgrA*, *TetO7*, *Pmin*) were amplified from pVG2.2 [37] using primer pairs TET-A-PoliC-F/TET-A-R and TET-B-F/TET-B-GFP-R (Table S3). This TET construct was fused to the constitutive promoter *PoliC*. *S. cerevisiae* was transformed with the obtained fragments as well as with the *Nco*I restricted plasmid pNAN-OGG [46], yielding pNAN-OTGG. To target pNAN-OTGG to the *DDR48* locus in *F. fujikuroi*, 2 kb of *DDR48* and its upstream sequence was amplified with primer pair TET_ddr_F/TET_ddr_R (Table S3) and cloned into *Eco*RI/*Sac*II restricted pNAN-OTGG, giving pTET. For fusion of *TF22* to the TET construct, the gene of interest was amplified with TF22_TET_F/TF22_TET_R (Table S3) and cloned into *Nco*I/*Not*I restricted pTET, yielding pTET::*TF22* (Figure S10a). The correct assembly of all overexpression vectors was verified by sequencing with primers listed in Table S3.

### 4.3. Fungal Transformations and Analysis of Transformants

Protoplast transformation of *F. fujikuroi* was carried out as previously described [50]. Replacement cassettes were amplified from the circular deletion vectors using primer pairs 5F/3R (Table S2). Then 10–30 µg of the (inducible) overexpression vectors pOE::*TF22* (Figure S9a), pOE::*TF23* (Figure S9b), pOE::*eqxD* (Figure S9c) and pTET::*TF22* (Figure S10a) was introduced in a circular manner. Selection of transformants was achieved with 100 µg/mL hygromycin B (Calbiochem, Darmstadt, Germany) and/or 100 µg/mL nourseothricin (Werner-Bioagents, Jena, Germany) depending on the resistance marker.

Homologous integration of the resistance cassettes and absence of WT genes was verified by Southern blot analysis as well as diagnostic PCR. Diagnostic PCRs for two or three independent transformants of Δ*PKS-NRPS1*, Δ*DA*, Δ*ER*, Δ*TF23,* and Δ*MFS-T* can be found in Figures S11–S15. Integration of the overexpression vector pOE::*TF22* was verified for two independent transformants of OE::*TF22* (Figure S9d; OE::*TF22* T9: ectopic integration; OE::*TF22* T13: in locus integration), the in locus integration of pOE::*TF23* was shown for two independent transformants of OE::*TF23* (Figure S9e) and the integration of pOE::*TF22* and pOE::*eqxD* was shown for three independent double mutants of OE::*TF22*/OE::*eqxD* (Figure S9f). Moreover, the in locus integration of pTET::*TF22* at *DDR48* was verified for two independent transformants of TET::*TF22*, Δ*PKS-NRPS1*/TET::*TF22*, Δ*DA*/TET::*TF22*, Δ*ER*/TET::*TF22*, Δ*TF23*/TET::*TF22* and Δ*MFS-T*/TET::*TF22*, respectively (Figure S10b–g).

### 4.4. Molecular Methods

Plasmid DNA from *S. cerevisiae* FY834 as well as *E. coli* Top10 F′ (Invitrogen, Darmstadt, Germany) was isolated with the NucleoSpin^®^ Plasmid Kit (Macherey-Nagel, Dueren, Germany). Genomic DNA from *F. fujikuroi* was extracted from lyophilized and ground mycelium following the protocol of Cenis [51]. For Southern blot analysis [52] of deletion mutants for ectopically integrated deletion cassettes, genomic DNA digested with an appropriate restriction enzyme (Thermo Fisher Scientific, Schwerte, Germany) was separated in a 1% (*w*/*v*) agarose gel and transferred to a nylon membrane (Nytran™ SPC, Whatman, Sanford, FL, USA) by downward alkali blotting [53]. Membranes were hybridized with ^32^P-labeled probes that were generated with the random oligomer-primer method [54], while 5′ or 3′ flanks (Table S2) were used as templates. Southern blot analyses of Δ*PKS-NRPS1*, Δ*DA*, Δ*ER*, Δ*TF23,* and Δ*MFS-T* deletion mutants can be found in Figures S11–S15. PCR amplification was performed by using BioTherm™ DNA Polymerase (GeneCraft, Luedinghausen, Germany), TaKaRa LA Taq^®^ DNA Polymerase (Takara Bio, Saint-Germain-en-Laye, France) or Phusion^®^ High-Fidelity DNA Polymerase (Finnzymes, Vantaa, Finland) according to the manufacturer’s instructions.

For RNA extraction from lyophilized and ground mycelium, the TRI Reagent™ (Sigma-Aldrich, Steinheim, Germany) was used. Total RNA (20 µg) was separated in a 1% (*w*/*v*) denaturating agarose gel [54] for expression analysis by Northern blot [55]. The RNA was transferred to a nylon membrane (Nytran™ SPC, Whatman, Sanford, FL, USA) and was hybridized with ^32^P-labeled probes as described above [54]. Probes, ca. 1 kb fragments of the genes of interest, were generated by PCR with primer pairs WT_F/WT_R for *FFUJ_02218*-*FFUJ_02225* (Table S2) as well as eqxD_OE_F/eqxD_OE_R for *F. heterosporum eqxD* (Table S3). For expression analysis by qRT-PCR, DNA in 1 µg of total RNA was first removed by digestion with DNase I (Thermo Fisher Scientific, Schwerte, Germany), and then was transcribed into cDNA applying oligo dT primers and SuperScript^®^ II Reverse Transcriptase (Invitrogen, Darmstadt, Germany) according to the manufacturer’s instructions. The qRT-PCR used the iQ SYBR Green Supermix (Bio-Rad, Muenchen, Germany) in a C1000 Touch™ Thermal Cycler with a CFX96™ Real-Time System (Bio-Rad, Muenchen, Germany). For quantifying transcript levels of the genes of interest (*PKS-NRPS1*, *DA*, *ER*, *TF22*, *TF23*, *MFS-T*) and of the constitutively expressed reference genes (*FFUJ_07710*, a GDP mannose transporter gene; *FFUJ_05652*, a related actin gene; *FFUJ_08398*, an ubiquitin gene), the primers listed in Table S4 were used. The annealing temperature was 60 °C and primer efficiencies were between 90% and 110%. Two technical and two biological replicates were carried out and the results were calculated with the ∆∆Ct-method [56].

### 4.5. Rice Virulence and Rice Germination Assays

The virulence assay on rice was performed with surface sterilized seedlings of *Oryza sativa* spp. *japonica* cv. Nipponbare following a slightly modified protocol described by Wiemann et al. [57]. Briefly, rice seeds without husks were incubated in 70% (*v*/*v*) ethanol (EtOH) for 3 × 1 min, then washed with sterile H_2_O three times, incubated in 6.5% (*v*/*v*) NaClO for 10 min and again washed with H_2_O three times. The surface sterilized seeds were kept on H_2_O agar (15 g/L) at 4 °C in the dark and in the absence of air (parafilm) for 3 days. Subsequently, germination took place at 28 °C and a 12 h light/12 h dark cycle for 5 days. Then 3 cm × 20 cm test tubes were filled with 4 cm vermiculite (Deutsche Vermiculite Daemmstoff GmbH, Sprockhoevel, Germany), two mycelial plugs (0.3 cm diameter) were added to each tube and covered with 1 cm vermiculite. Test tubes were watered with 10 mL Gamborg B5 solution (3.16 g/L; Duchefa Biochemie, Haarlem, The Netherlands), and the germinated rice seedlings were placed on top. As positive control, 100 parts per million (ppm) gibberellic acid GA_3_ was added; non-treated rice seedlings served as negative control (H_2_O control). The length between the first and the second internode was measured 7 days-post-infection (28 °C, 12 h light/12 h dark).

For the rice germination assay, 50 surface sterilized and cold-treated seedlings were incubated with fungal suspension or H_2_O as control, respectively, for 16 h. For generating the fungal suspension, three mycelial plugs (0.6 cm diameter) were ground in 0.5 mL sterile H_2_O, adjusting a final volume of 30 mL with H_2_O. The treated seeds were germinated on Whatman paper (Whatman, Sanford, FL, USA) at 28 °C and a 12 h light/12 h dark cycle for 6 days and watered with sterile H_2_O when needed.

### 4.6. Cytotoxicity Assay

For cytotoxicity studies, human hepatocellular carcinoma cells (Hep G2, ACC 180; DSMZ, Braunschweig, Germany) and the kit CCK-8 (Dojindo Laboratories, Tokyo, Japan) were used. The cells were seeded on 96-well microplates at a density of 1 × 10^4^ cells/well. After 24 h stabilization, the cells were treated with serum-free medium and 24 h later, the cells were incubated for 48 h with 0.1–50 µM trichosetin or equisetin (Santa Cruz Biotechnology, Santa Cruz, CA, USA; purity >99%). As negative control, 1% (*v*/*v*) MeOH was added and as positive control, Hep G2 cells were incubated with 10 µM of the trichothecene mycotoxin T-2. Studies were performed in triplicate with cells from three independent passages. After 48 h of exposure, the dye solution (water-soluble tetrazolium salt, WST-8) was added to the cells, followed by incubation at 37 °C for 90 min. The WST-8 reduction by cellular dehydrogenases from viable cells produces a water-soluble formazan dye and increases the absorbance at λ = 450 nm which was measured with a microplate reader (Tecan Infinite^®^ M200 PRO, Salzburg, Austria). IC_50_ values were calculated with OriginPro 2016 (OriginLab Corporation, Northampton, MA, USA) after *log*-transformation of the concentration values and sigmoidal fit with the dose response function.

### 4.7. Isolation of Trichosetin

The culture filtrate of OE:*:TF22* T9 was extracted five times with pentane (1:1, *v*/*v*) and evaporated to dryness afterwards. The residue was dissolved in 50% (*v*/*v*) acetonitrile (ACN) with 0.1% (*v*/*v*) formic acid (FA) and applied to a Strata C18-E (10 g/60 mL) SPE column (Phenomenex, Aschaffenburg, Germany). This SPE column was flushed with 50 mL ACN and 50 mL 50% (*v*/*v*) ACN + 0.1% (*v*/*v*) FA under vacuum before applying the extract. Metabolites were eluted from the column in fractions of 50%, 60%, 70%, and 100% (*v*/*v*) ACN + 0.1% (*v*/*v*) FA. The fraction with 70% (*v*/*v*) ACN + 0.1% (*v*/*v*) FA was evaporated to dryness, and 500 µL H_2_O was applied for a washing step. The received slurry was put in an ultrasonic bath and centrifuged with 2950 g at room temperature. The supernatant was discarded; the residue was freeze-dried to obtain trichosetin with 99.5% purity (Figure S16d). Purity measurements of trichosetin were performed on a HPLC-evaporative light scattering detector (ELSD) with a LC-20AT system (Shimadzu, Duisburg, Germany). The column used was a 150 mm × 3.0 mm i.d., 5 µm, Zorbax Extend-C18 column (Agilent Technologies, Boeblingen, Germany) with H_2_O as solvent A and ACN as solvent B. The temperature of the ELSD was set to 40 °C, and 2.5 bar of pressurized air was used. The gradient of the ELSD measurement started with 10% B with 1 mL/min, holding these conditions for 3 min. In 27 min, the gradient rose up to 100% B, followed by 10 min 100% B. Finally, the column was equilibrated for 5 min at 10%.

### 4.8. Physico-Chemical Properties of Trichosetin

The UV spectra were obtained with a Jasco V-750 spectrophotometer (Jasco Labor- und Datentechnik, Groß-Umstadt, Germany), the circular dichroism (CD) spectrum was measured with a Jasco J-600 CD spectrometer. The spectra can be found in Figure S16a–c.

UV λ_max_ (MeOH) nm: 203, 256, 293; UV λ_max_ (ACN) nm: 244, 284. The molar extinction coefficient (ε λ_max_ (ACN) L/(mol × cm) = 9242 (284 nm)) was used to determine the concentration of further isolates.

CD λ_max_ (mol CD): 320 (+3.6), 290 (−12), 250 (−0.5), 229 (−2.2), 203 (−55). Compared to the literature [28], the CD is shifted, but the CD curve form is alike. The CD data of equisetin and *N*-demethylophiosetin are more similar to our data obtained for trichosetin [41].

Nuclear magnetic resonance (NMR) spectra were recorded in MeOH-d_4_ on an Agilent DD2 600 MHz spectrometer (Agilent Technologies, Boeblingen, Germany) equipped with a cold probe. The signals are reported in ppm and are referenced to tetramethylsilan. ^1^H NMR, ^13^C NMR, gHMBC, gCOSY, NOESY data and a comparison with the literature can be found in Tables S5 and S6; HPLC-HRMS *m/z* [M + H]^+^ calculated for C_21_H_30_O_4_N^+^: 360.2169; found: 360.2174.

### 4.9. HPLC Analysis of Trichosetin and Equisetin

All solvents and chemicals were used in analytical grade from Sigma-Aldrich (Steinheim, Germany), VWR (Darmstadt, Germany) or Thermo Fisher Scientific (Schwerte, Germany).

The supernatant of 7-day-old ICI + 60 mM glutamine cultures was filtered using 0.45 µm membrane filters (BGB Analytik, Schlossboeckelheim, Germany) and analyzed via HPLC-HRMS or HPLC-MS/MS as described below. Metabolite extraction from fungal mycelium or from rice plants was performed with a modified method described by Niehaus et al. [12]. Briefly, 0.1 g of washed and lyophilized mycelium was mixed with 1.5 mL ethyl acetate:MeOH:dichlormethane (3:2:1, *v*/*v*) for 2 h. 0.5 mL of the extract was evaporated to dryness and taken up in 0.2 mL 50% (*v*/*v*) ACN. Furthermore, ca. 0.05 g of lyophilized and ground rice plants was mixed with 1.5 mL ethyl acetate:MeOH:dichlormethane (3:2:1*, v/v*) for 1 h, whereupon 0.75 mL of the extract was evaporated and taken up in 0.2 mL 50% (*v*/*v*) ACN. The extracts were vortexed and put in an ultrasonic bath for 10 min. The samples were then centrifuged with 1600 g before further dilution as described below.

The HPLC-HRMS measurements were carried out as follows: the culture filtrates of OE::*TF22* T9 and the WT for the identification of oxidized trichosetin derivatives were analyzed as described by Arndt et al. [7]. For the measurements of Δ*DA*/TET::*TF22* samples as well as OE::*TF22* T9 and T13 comparison, a different HPLC setup was used: the Accela system was replaced with a Shimadzu HPLC system (LC-20AD HPLC, SIL*-*20ACXE (autosampler), CTO-10ASvp (oven), SPD-M20A (PDA detector) and CBM-20A (controller); Shimadzu, Duisburg, Germany). A Nucleodur C18 HTec column (100 mm × 2.0 mm, 3 µm; Macherey-Nagel, Dueren, Germany) was used at 40 °C with MeOH + 0.01% (*v*/*v*) FA as eluent A and H_2_O + 0.01% (*v*/*v*) FA as eluent B. The gradient started with 65% A with a flow rate of 0.25 mL/min. It rose up to 100% A in 30 min, holding this condition for 5 min. Afterwards, the column was re-equilibrated for 5 min at 65% A. The settings of the LTQ Orbitrap XL (Thermo Fisher Scientific, Schwerte, Germany) were applied as described by Arndt et al. [7], but collision-induced dissociation (CID) was chosen to compare the putative stereoisomers in Δ*DA*/TET::*TF22*. Therefore, 40% normalized collision energy (NCE) for *m/z* 360.22 as precursor ion was applied, and the highest and second highest product ions were fragmented with 15% NCE again. The received fragmentation patterns can be found in Table S1.

For trichosetin and equisetin analysis, HPLC-MS/MS was applied, with ochratoxin A (OTA) as internal standard (isolated in previous work; [58]). Therefore, 10 µL of the culture filtrate or the above described mycelium extracts was mixed with 80 µL 50% (*v*/*v*) ACN and 10 µL OTA standard solution (1 µg/mL in ACN). For analysis of in planta metabolite levels, 10 µL OTA standard solution was added to 90 µL of the extract.

For semi-quantification of trichosetin and equisetin, an external calibration curve was generated, with the following concentrations of trichosetin or equisetin: 0.15 µg/mL, 0.25 µg/mL, 0.50 µg/mL, 1.00 µg/mL, 1.50 µg/mL, and 10.0 µg/mL in 50% (*v*/*v*) ACN. OTA was added to obtain a final concentration of 0.1 µg/mL as internal standard, as described for the preparation of the samples. Peak areas of the analytes were divided by the corresponding peak area of OTA and a calibration curve was generated out of the received values (Figure S17). Samples were analyzed alike and their concentrations (µg/mL liquid culture) were calculated with the function of the calibration curve. The absolute amount of both analytes in the cultures (30 or 100 mL) was referenced to the respective fungal biomass (dry weight) of the flasks.

Concerning HPLC-MS/MS measurements, the flow rate of the Agilent 1200 series HPLC system (Agilent Technologies, Boeblingen, Germany) was set to 250 µL/min, with ACN + 1% (*v*/*v*) formic acid as eluent A and H_2_O + 1% (*v*/*v*) FA as eluent B. A Luna C8 column (2 mm × 150 mm, 3 µm; with fitting pre-column; Phenomenex, Aschaffenburg, Germany) was used at 40 °C. The gradient started with 55% A, stayed at this level for 3 min and rose up to 100% A in 7 min. After 5 min at 100% A, the initial setting (55% A) was applied again, holding this condition for an additional 3 min. The MS/MS parameters for the API 3200 mass spectrometer (Applied Biosystems, Darmstadt, Germany) were as follows: for electrospray ionization, the ion spray voltage was set to +4500 V. The curtain gas was set to 30 psi, the collision gas to 10 psi, the nebulizer gas to 35 psi and the auxiliary gas to 45 psi at 350 °C. All dwell times were set to 20 ms, the cell exit potential was set to 5 V and the entrance potential was set to 5 V for all multiple reaction monitoring (MRM) transitions. For trichosetin analysis (retention time 9.56 min), the following parameters and MRM transitions were used: the declustering potential (DP) was set to 33 V and the collision cell entrance potential (CEP) was set to 15 V; quantifier transition: *m/z* 360→175 (collision energy (CE) 20 V); qualifier transition 1: *m/z* 360→81 (CE 46 V); qualifier transition 2: *m/z* 360→119 (CE 35 V). For equisetin analysis (retention time 10.49 min), the following parameters and MRM transitions were used: DP 45 V, CEP 20 V; quantifier transition: *m/z* 374→175 (CE 20 V); qualifier transition 1: *m/z* 374→140 (CE 55 V); qualifier transition 2: *m/z* 374→81 (CE 45 V). The internal standard OTA was analyzed as follows: retention time 4.29 min; DP 32 V, CEP 20 V; quantifier transition: *m/z* 404→239 (CE 30 V); qualifier transition 1: *m/z* 404→102 (CE 95 V); qualifier transition 2: *m/z* 404→221 (CE 47 V). A divert valve was used to discard the first and the last 3 min of the run.

## Supplementary Materials

The following are available online at www.mdpi.com/2072-6651/9/4/126/s1, Figure S1: HPLC-HRMS extracted ion chromatograms (XICs) of trichosetin (*m*/*z* [M + H]^+^ 360.2169, Δppm = 5; retention time 16.31 min) for OE::*TF22* transformants T9 and T13 in comparison to the wild type (WT). The strains were grown in liquid culture for 7 days. The y axis is not identical for all XICs to ensure visibility of the analyte in the transformant T13, Figure S2: Phenotypic analysis of TET::*TF22* double mutants. The wild type (WT), TET::*TF22* and indicated double mutants were grown on solid CM for 3 days. The medium was supplemented with 0, 10 or 50 µg/mL doxycycline (Dox) for induction of transcription factor (TF) gene expression. T, transformant, Figure S3: Real-time expression analysis of TET::*TF22* double mutants. The wild type (WT) and two independent transformants (T) of TET::*TF22* single and double mutants were grown on solid CM for 3 days. The medium was supplemented with 0, 10 or 50 µg/mL doxycycline (Dox) for induction of transcription factor (TF) gene expression. Total RNA was isolated from the harvested mycelium, transcribed into cDNA and the relative expression (RE) of *TF22* was analyzed using the ΔΔCt method. Error bars (±standard deviation) originate from a technical replicate and expression of TET::*TF22* T10, 50 µg/mL Dox was arbitrarily set to 1, Figure S4: HPLC-HRMS analysis of the Δ*DA*/TET::*TF22* double mutant. Shown are the extracted ion chromatograms of trichosetin (*m/z* [M + H]^+^ 360.2169, Δppm = 5). TET::*TF22* and Δ*DA*/TET::*TF22* transformants (T) were grown in liquid culture for 2 days, then transcription factor (TF) gene expression was induced with 50 µg/mL doxycycline for an additional 5 days, Figure S5: Trichosetin plate assay. (**a**) The wild type (WT), Δ*MFS-T* and Δ*TF23* transformants (T) were grown on solid CM supplemented with 0, 5 or 10 µg/mL trichosetin for 4 days. (**b**) The cultivation was done in triplicate and average colony diameters are shown, Figure S6: Rice germination assay using H_2_O (negative control), the *F. fujikuroi* wild type (WT) as well as one transformant (T) of OE::*TF22*. (**a**) Surface sterilized rice seedlings were treated with H_2_O or fungal suspension for 16 h, then seedlings were incubated for 6 days in the presence of a 12 h light/12 h dark cycle to germinate. Arrows indicate *bakanae* symptoms. (**b**) Out of 50 seedlings, the germination of only shoot or shoot + root was counted and related to the H_2_O control which was set to 100%, Figure S7: CCK-8 assay on Hep G2 cells applying 0.1–50 µM equisetin or trichosetin. 1% methanol (MeOH) and 10 µM T-2 toxin served as negative and positive control, respectively. The data represent mean values (±standard deviation). The significance indicated refers to the solvent-treated control (1% MeOH) calculated with an unequal variances *t*-test; *** statistically highly significant (*p* ≤0.001), Figure S8: HPLC-HRMS extracted ion chromatograms of *m/z* [M + H]^+^ 376.2118 (calculated for hydroxy- or keto-trichosetin, Δppm = 5) for the wild type (WT) and OE::*TF22* T9. The strains were grown in liquid culture for 7 days, Figure S9: Overexpression of *TF22* and *TF23* via constitutive *PoliC* promoter from *A. nidulans* as well as overexpression of *eqxD* via constitutive *PglnA* promoter from *F. fujikuroi*. (**a**) The full-length gene *TF22* including 244 bp of the native terminator sequence (*T*) was cloned into *Nco*I/*Sac*II restricted pNDH-OGG conferring hygromycin B resistance (*hphR*). (**b**) The first 1.2 kb of *TF23* was cloned into *Nco*I/*Sac*II restricted pNDH-OGG conferring hygromycin B resistance (*hphR*). (**c**) The full-length gene *eqxD* from *F. heterosporum* was cloned into *Nco*I/*Not*I restricted pNAN-GGT conferring nourseothricin resistance (*natR*). (**d**) The integration of pOE::*TF22* in two independent transformants (T) was checked using primer pairs PoliC_Seq_F2/TF22_OE_R (1.82 kb) and PoliC_Seq_F2/TF22_OE_diag (1.86 kb). OE::*TF22* T9: ectopic integration; OE::*TF22* T13: in locus integration. (**e**) The in locus integration of pOE::*TF23* in two independent transformants (T) was checked using primer pair PoliC_Seq_F2/02223_WT_R (1.74 kb). (**f**) The integration of pOE::*TF22* and pOE::*eqxD* in three independent transformants (T) was checked using primer pairs PoliC_Seq_F2/TF22_OE_R (1.82 kb) and eqxD_OE_F/eqxD_OE_R (1.18 kb), respectively. The *F. fujikuroi* wild type (WT) was used as control. λ, λ/*Hin*dIII; M: GeneRuler DNA Ladder Mix, Figure S10: Inducible overexpression of *TF22*. (**a**) The full-length gene *TF22* was cloned into *Nco*I/*Not*I restricted pTET conferring nourseothricin resistance (*natR*). For pTET, the TET construct was fused to the constitutive *PoliC* promoter from *A. nidulans*, which encodes the tetracycline-dependent transactivator rtTA2^S^-M2 and furthermore, harbors the tetracycline-responsive element TRE. 2 kb of *DDR48* and its upstream sequence targets pTET to the constitutively expressed *DDR48* locus. pTET::*TF22* was transformed into all relevant genetic backgrounds, (**b**) the *F. fujikuroi* wild type (WT), (**c**) Δ*PKS-NRPS1*, (**d**), Δ*DA*, (**e**) Δ*ER*, (**f**) Δ*TF23* and (**g**) Δ*MFS-T*. The presence of pTET and the correct in locus integration in two independent transformants (T) was checked using primer pairs TET_Seq_F/02222_WT_R (1.50 kb) and Tgluc_hiF/TET_ddr_diag_R (2.10 kb), respectively. The WT or the respective deletion mutant was used as control. M: GeneRuler DNA Ladder Mix, Figure S11: Verification of Δ*PKS-NRPS1* deletion mutants by diagnostic PCR and Southern blot. (**a**) Deletion via homologous recombination with the hygromycin B resistance cassette (*hphR*) was underlined with the amplification of 5′ (trpC_T/02219_5diag) and 3′ (trpC_P2/02219_3diag) flanks but no amplification of wild-type (WT; 02219_WT_F/02219_WT_R) signal for two independent transformants (T). (**b**) For analyzing ectopic integration of deletion constructs, genomic DNA of transformants and WT was digested with *Stu*I and the 3′ flank was applied for probing. (**c**) Detected signals match the expected 2.07 kb for the WT as well as 5.61 kb for Δ*PKS-NRPS1*. λ: λ/*Hin*dIII, M: GeneRuler DNA Ladder Mix, Figure S12: Verification of Δ*DA* deletion mutants by diagnostic PCR and Southern blot. (**a**) Deletion via homologous recombination with the hygromycin B resistance cassette (*hphR*) was underlined with the amplification of 5′ (trpC_T/02220_5diag) and 3′ (trpC_P2/02220_3diag) flanks but no amplification of wild-type (WT; 02220_WT_F/02220_WT_R) signal for three independent transformants (T). (**b**) For analyzing ectopic integration of deletion constructs, genomic DNA of transformants and WT was digested with *Eco*RI and the 3′ flank was applied for probing. (**c**) Detected signals match the expected 7.72 kb for the WT as well as 3.38 kb for Δ*DA*. λ: λ/*Hin*dIII, M: GeneRuler DNA Ladder Mix, Figure S13: Verification of Δ*ER* deletion mutants by diagnostic PCR and Southern blot. (**a**) Deletion via homologous recombination with the hygromycin B resistance cassette (*hphR*) was underlined with the amplification of 5′ (trpC_T/02221_5diag) and 3′ (trpC_P2/02221_3diag) flanks but no amplification of wild-type (WT; 02221_WT_F/02221_WT_R) signal for three independent transformants (T). (**b**) For analyzing ectopic integration of deletion constructs, genomic DNA of transformants and WT was digested with *Sca*I and the 5′ flank was applied for probing. (**c**) Detected signals match the expected 10.66 kb for the WT as well as 5.26 kb for Δ*ER*. λ: λ/*Hin*dIII, M: GeneRuler DNA Ladder Mix, Figure S14: Verification of Δ*TF23* deletion mutants by diagnostic PCR and Southern blot. (**a**) Deletion via homologous recombination with the hygromycin B resistance cassette (*hphR*) was underlined with the amplification of 5′ (trpC_T/02223_5diag) and 3′ (trpC_P2/02223_3diag) flanks but no amplification of wild-type (WT; 02223_WT_F/02223_WT_R) signal for two independent transformants (T). (**b**) For analyzing ectopic integration of deletion constructs, genomic DNA of transformants and WT was digested with *Hin*dIII and the 5′ flank was applied for probing. (**c**) Detected signals match the expected 2.87 kb for the WT as well as 4.43 kb for Δ*TF23*. λ: λ/*Hin*dIII, M: GeneRuler DNA Ladder Mix, Figure S15: Verification of Δ*MFS-T* deletion mutants by diagnostic PCR and Southern blot. (**a**) Deletion via homologous recombination with the hygromycin B resistance cassette (*hphR*) was underlined with the amplification of 5′ (trpC_T/02224_5diag) and 3′ (trpC_P2/02224_3diag) flanks but no amplification of wild-type (WT; 02224_WT_F/02224_WT_R) signal for three independent transformants (T). (**b**) For analyzing ectopic integration of deletion constructs, genomic DNA of transformants and WT was digested with *Sca*I and the 5′ flank was applied for probing. (**c**) Detected signals match the expected 5.26 kb for the WT as well as 2.97 kb for Δ*MFS-T*. λ: λ/*Hin*dIII, M: GeneRuler DNA Ladder Mix, Figure S16: Analysis of physico-chemical properties and purity of trichosetin. (**a**) UV-spectrum of trichosetin in acetonitrile. (**b**) UV-spectrum of trichosetin in methanol. (**c**) Molar CD spectrum of trichosetin in methanol. (**d**) HPLC-ELSD chromatogram of trichosetin, retention time 21.27 min, Figure S17: Calibration curve of the semi-quantitative analysis of trichosetin and equisetin, respectively. The analysis was done with HPLC-MS/MS, and OTA was used as internal standard (IS). The corresponding function of the calibration curves as well as the Pearson correlation coefficient R^2^ are given adjacent to the names of the analytes, Table S1: HPLC-HRMS-CID measurement of trichosetin and the two putative stereoisomers in Δ*DA*/TET::*TF22*. The precursor ion *m/z* 360.22 was fragmented with 40% normalized collision energy (NCE). The highest and second highest fragment ions were fragmented again with 15% NCE. The “?” in the table indicate that the ppm deviation of calculated to measured exact mass was higher than 5 ppm, Table S2: Primer sequences used for the generation of deletion constructs, verification of their homologous integration as well as for probe generation. Introduced overhangs required for yeast recombinational cloning are underlined, Table S3: Primer sequences used for the generation and analysis of constitutive and inducible overexpression vectors. Introduced overhangs required for yeast recombinational cloning are underlined, Table S4: Primer sequences used for expressional analysis by quantitative real-time PCR. Reference genes: *GMT*, GDP mannose transporter gene; *RAC*, related actin gene; *UBI*, ubiquitin gene, Table S5: NMR spectra of trichosetin in MeOH-d_4_, measured with a 600 MHz NMR-spectrometer and referenced to tetramethylsilane. Signals are given in ppm. The number of the corresponding carbon atoms (No. of C)le S is similar to that reported by Marfori et al. [28]. Multiple proton signals are divided by a semicolon, Table S6: NMR spectra of trichosetin in MeOH-d_4_, measured with a 600 MHz NMR-spectrometer and referenced to tetramethylsilane, in comparison to NMR spectra found in the literature. Signals are given in ppm. The number of the corresponding carbon atoms (No. of C) is similar to that reported by Marfori et al. [28]. Multiple proton signals are divided by a semicolon.
