# Supplementary material for: Establishment of the Inducible Tet-On System for the Activation of the Silent Trichosetin Gene Cluster in Fusarium fujikuroi
Source: Toxins (Basel). 2017 Apr 5;9(4):126. doi: 10.3390/toxins9040126 (PMC5408200; doi:10.3390/toxins9040126)
Supplement: Supplementary file 1 [file toxins-09-00126-s001.pdf]

Supplementary Materials: Establishment of the Inducible Tet-on System for the Activation of the Silent Trichosetin Gene Cluster in *Fusarium fujikuroi*

Slavica Janevska, Birgit Arndt, Leonie Baumann, Lisa Helene Apken, Lucas Maciel Mauriz Marques, Hans-Ulrich Humpf, Bettina Tudzynski

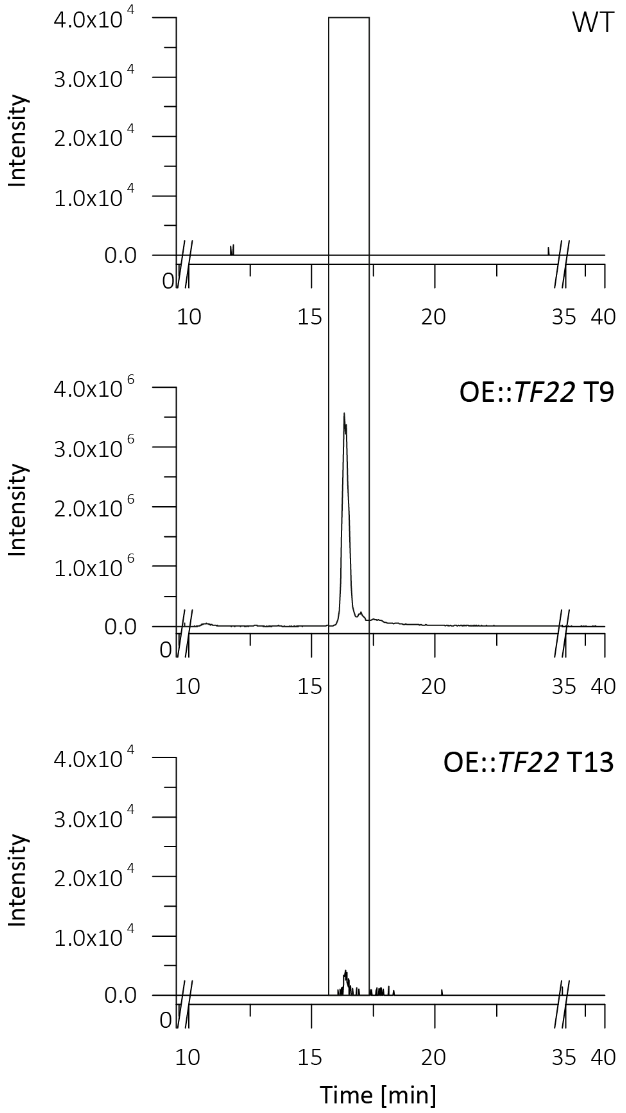

Figure S1. HPLC-HRMS extracted ion chromatograms (XICs) of trichosetin (*m*/*z* [M + H]$^+$ 360.2169, Δppm = 5; retention time 16.31 min) for OE::*TF22* transformants T9 and T13 in comparison to the wild type (WT). The strains were grown in liquid culture for 7 days. The y axis is not identical for all XICs to ensure visibility of the analyte in the transformant T13.

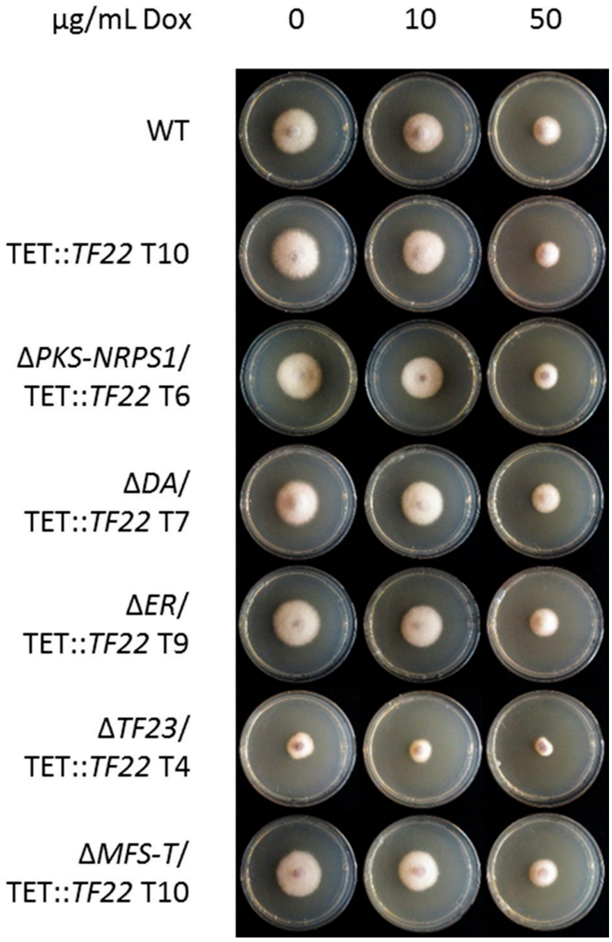

Figure S2. Phenotypic analysis of TET::*TF22* double mutants. The wild type (WT), TET::*TF22* and indicated double mutants were grown on solid CM for 3 days. The medium was supplemented with 0, 10 or 50 µg/mL doxycycline (Dox) for induction of transcription factor (TF) gene expression. T, transformant.

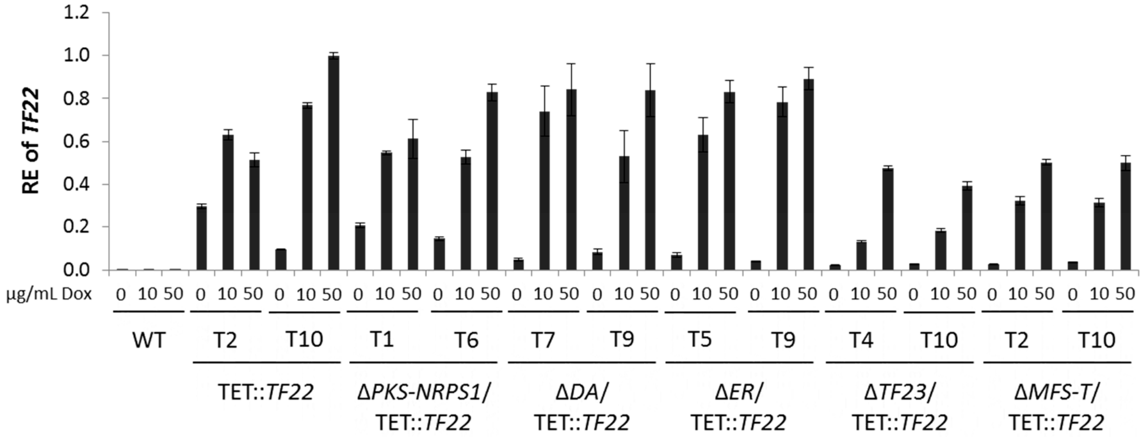

Figure S3. Real-time expression analysis of TET::*TF22* double mutants. The wild type (WT) and two independent transformants (T) of TET::*TF22* single and double mutants were grown on solid CM for 3 days. The medium was supplemented with 0, 10 or 50 µg/mL doxycycline (Dox) for induction of transcription factor (TF) gene expression. Total RNA was isolated from the harvested mycelium, transcribed into cDNA and the relative expression (RE) of *TF22* was analyzed using the ΔΔCt method. Error bars (±standard deviation) originate from a technical replicate and expression of TET::*TF22* T10, 50 µg/mL Dox was arbitrarily set to 1.

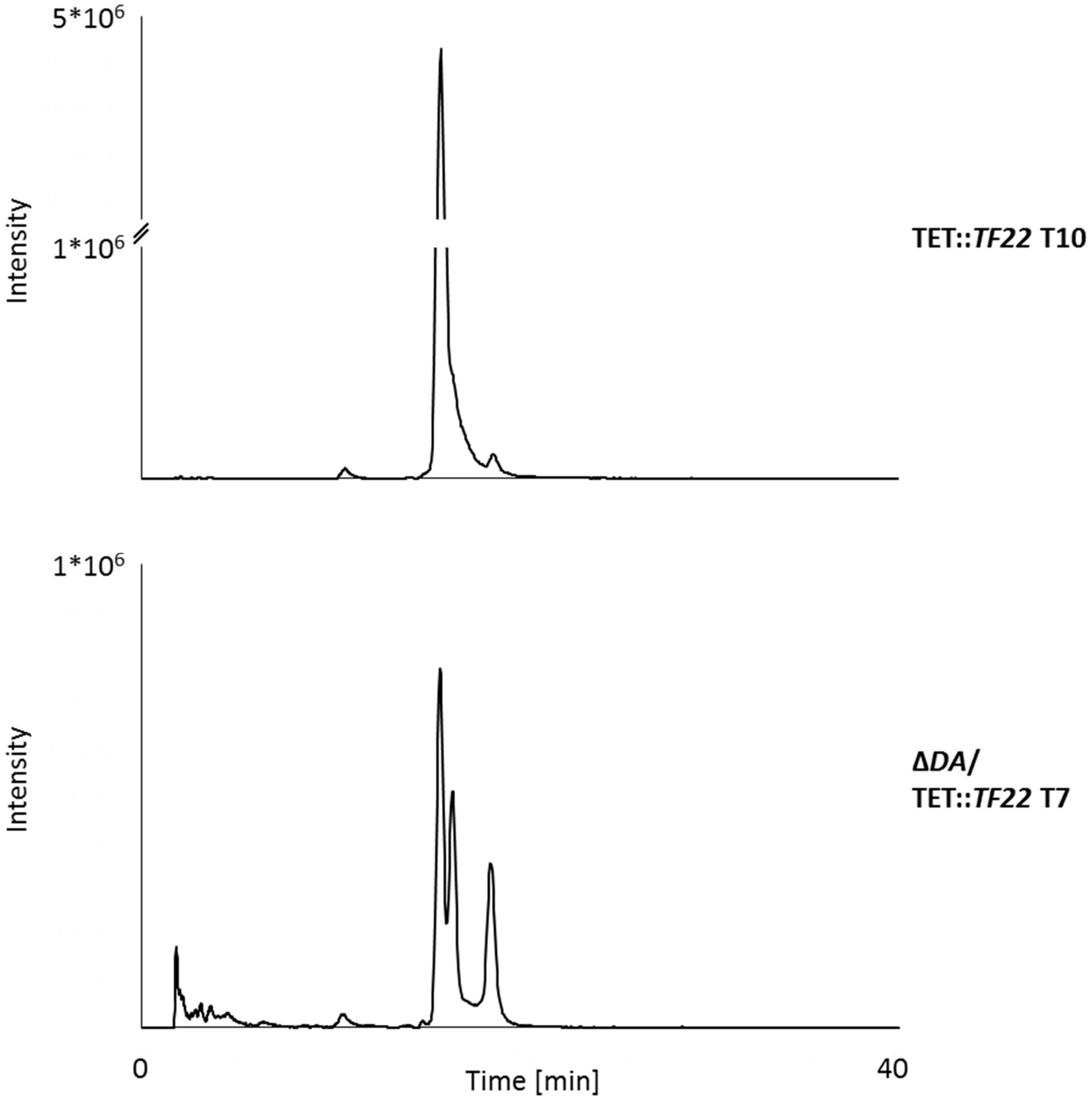

Figure S4. HPLC-HRMS analysis of the Δ*DA*/TET::*TF22* double mutant. Shown are the extracted ion chromatograms of trichosetin (*m*/*z* $[M + H]^+$ 360.2169, Δppm = 5). TET::*TF22* and Δ*DA*/TET::*TF22* transformants (T) were grown in liquid culture for 2 days, then transcription factor (TF) gene expression was induced with 50 μg/mL doxycycline for an additional 5 days.

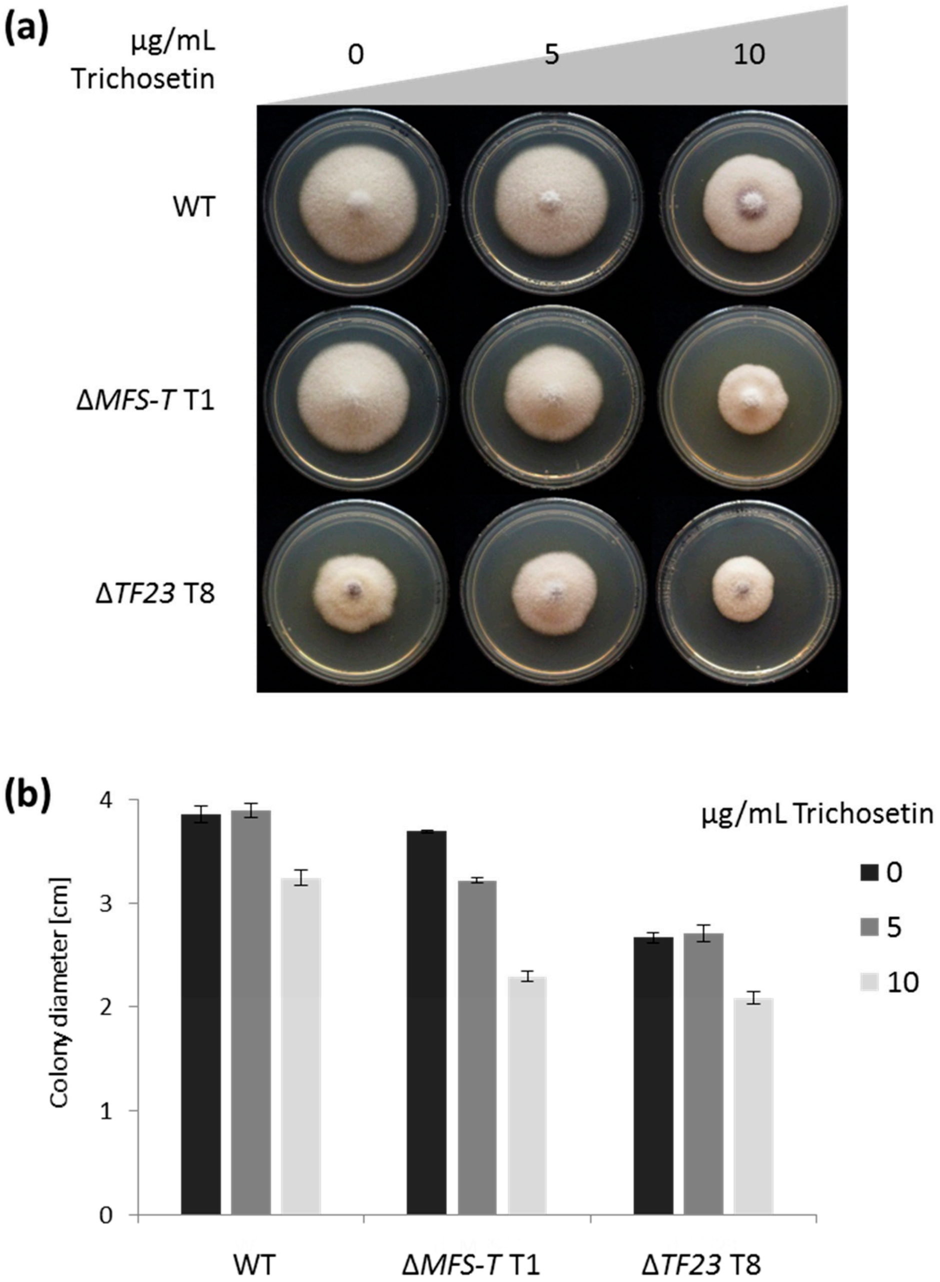

Figure S5. Trichosetin plate assay. (**a**) The wild type (WT), Δ*MFS-T* and Δ*TF23* transformants (T) were grown on solid CM supplemented with 0, 5 or 10 µg/mL trichosetin for 4 days. (**b**) The cultivation was done in triplicate and average colony diameters are shown.

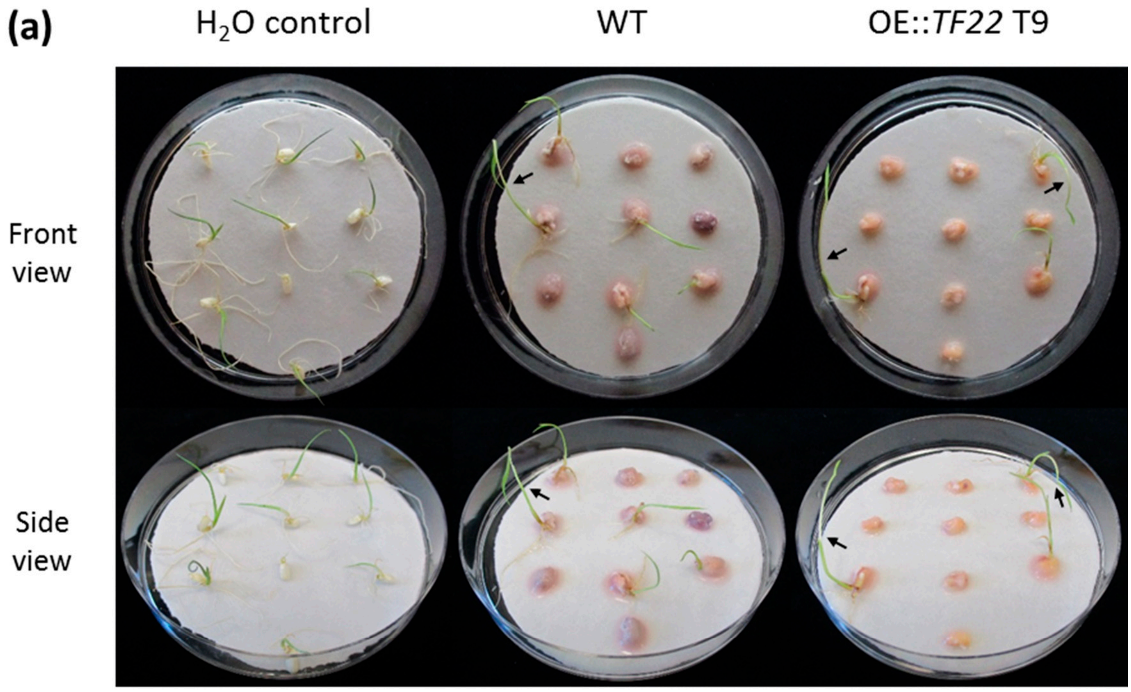

(b)

Strain	Shoot	Shoot + root
H_2O control	41 (100%)	41 (100%)
WT	31 (76%)	18 (44%)
OE::*TF22* T9	26 (63%)	10 (24%)

Figure S6. Rice germination assay using H_2O (negative control), the *F. fujikuroi* wild type (WT) as well as one transformant (T) of OE::*TF22*. (**a**) Surface sterilized rice seedlings were treated with H_2O or fungal suspension for 16 h, then seedlings were incubated for 6 days in the presence of a 12 h light/12 h dark cycle to germinate. Arrows indicate *bakanae* symptoms. (**b**) Out of 50 seedlings, the germination of only shoot or shoot + root was counted and related to the H_2O control which was set to 100%.

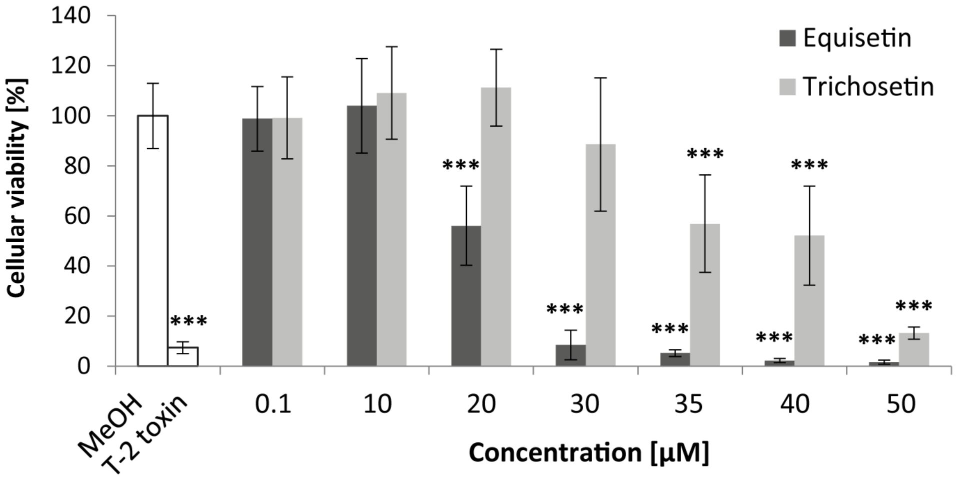

Figure S7. CCK-8 assay on Hep G2 cells applying 0.1-50 µM equisetin or trichosetin. 1% methanol (MeOH) and 10 µM T-2 toxin served as negative and positive control, respectively. The data represent mean values (±standard deviation). The significance indicated refers to the solvent-treated control (1% MeOH) calculated with an unequal variances *t*-test; *** statistically highly significant ($p \leq 0.001$).

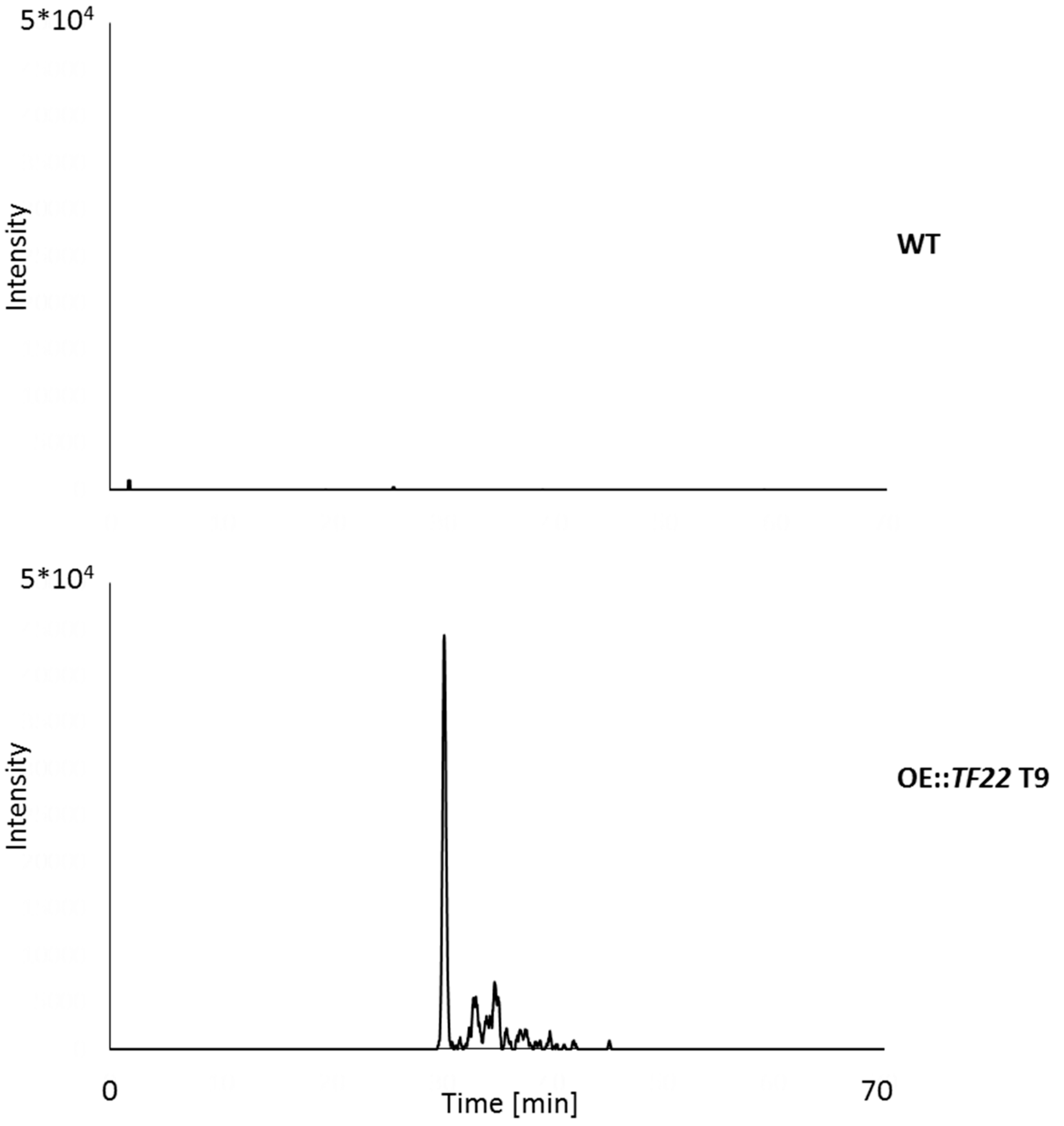

Figure S8. HPLC-HRMS extracted ion chromatograms of *m*/*z* $[M + H]^+$ 376.2118 (calculated for hydroxy- or keto-trichosetin, Δppm = 5) for the wild type (WT) and OE::*TF22* T9. The strains were grown in liquid culture for 7 days.

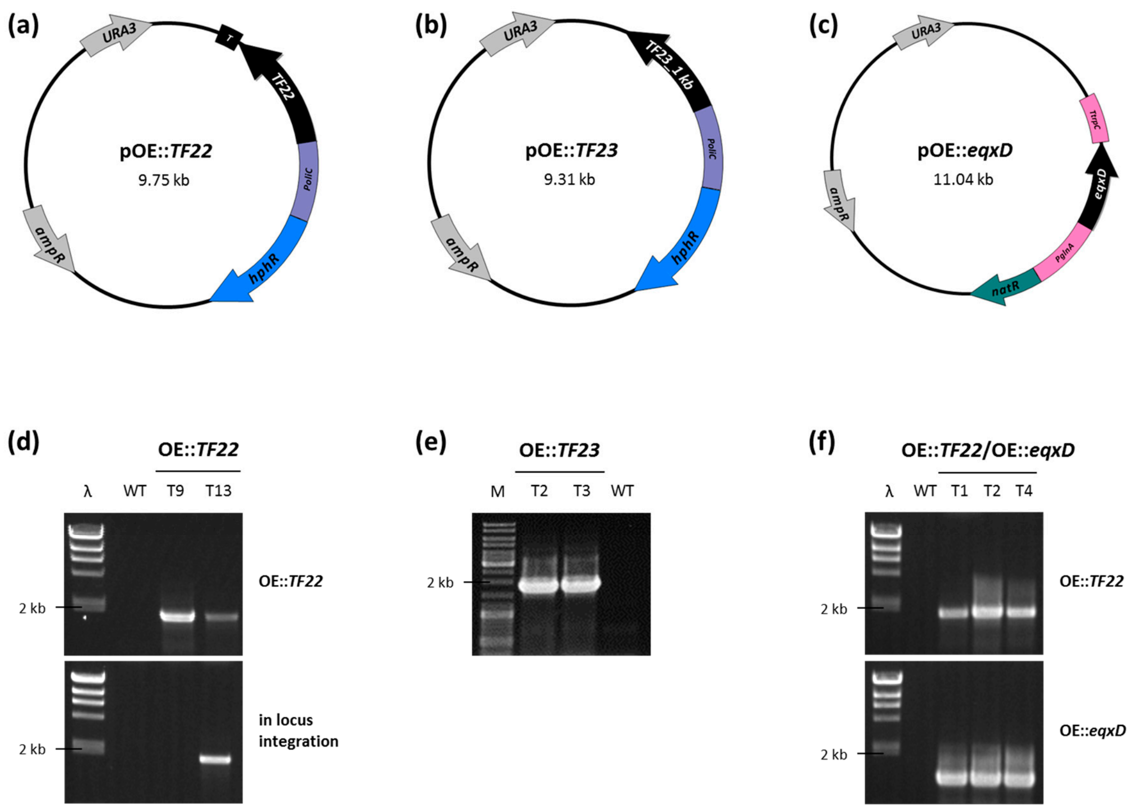

Figure S9. Overexpression of *TF22* and *TF23* via constitutive *PoliC* promoter from *A. nidulans* as well as overexpression of *eqxD* via constitutive *PglnA* promoter from *F. fujikuroi*. (**a**) The full-length gene *TF22* including 244 bp of the native terminator sequence (*T*) was cloned into *Nco*I/*Sac*II restricted pNDH-OGG conferring hygromycin B resistance (*hphR*). (**b**) The first 1.2 kb of *TF23* was cloned into *Nco*I/*Sac*II restricted pNDH-OGG conferring hygromycin B resistance (*hphR*). (**c**) The full-length gene *eqxD* from *F. heterosporum* was cloned into *Nco*I/*Not*I restricted pNAN-GGT conferring nourseothricin resistance (*natR*). (**d**) The integration of pOE::*TF22* in two independent transformants (T) was checked using primer pairs PoliC_Seq_F2/TF22_OE_R (1.82 kb) and PoliC_Seq_F2/TF22_OE_diag (1.86 kb). OE::*TF22* T9: ectopic integration; OE::*TF22* T13: in locus integration. (**e**) The in locus integration of pOE::*TF23* in two independent transformants (T) was checked using primer pair PoliC_Seq_F2/02223_WT_R (1.74 kb). (**f**) The integration of pOE::*TF22* and pOE::*eqxD* in three independent transformants (T) was checked using primer pairs PoliC_Seq_F2/TF22_OE_R (1.82 kb) and eqxD_OE_F/eqxD_OE_R (1.18 kb), respectively. The *F. fujikuroi* wild type (WT) was used as control. λ, λ/*Hin*dIII; M: GeneRuler DNA Ladder Mix.

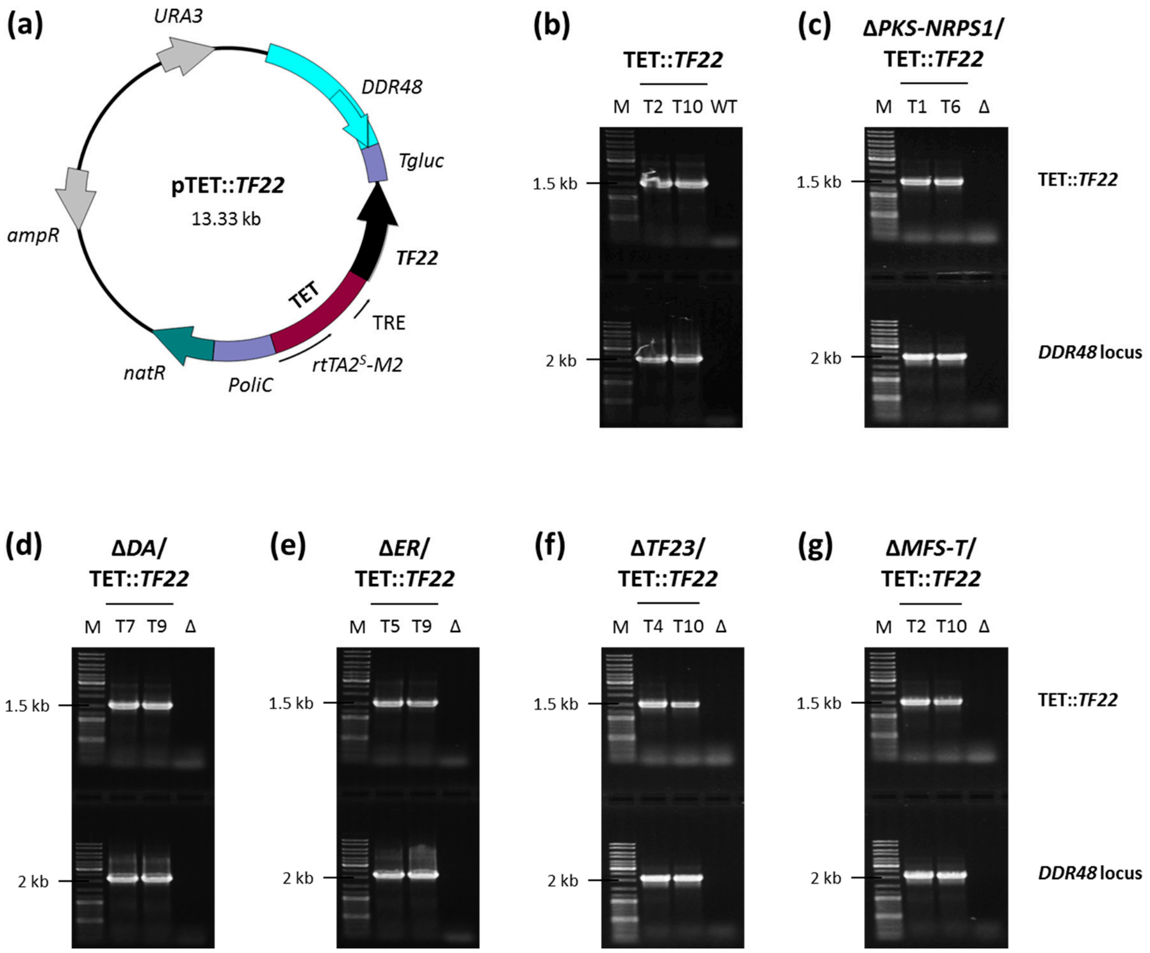

Figure S10. Inducible overexpression of *TF22*. (**a**) The full-length gene *TF22* was cloned into *Nco*I/*Not*I restricted pTET conferring nourseothricin resistance (*natR*). For pTET, the TET construct was fused to the constitutive *PoliC* promoter from *A. nidulans*, which encodes the tetracycline-dependent transactivator rtTA2^S-M2 and furthermore, harbors the tetracycline-responsive element TRE. 2 kb of *DDR48* and its upstream sequence targets pTET to the constitutively expressed *DDR48* locus. pTET::*TF22* was transformed into all relevant genetic backgrounds, (**b**) the *F. fujikuroi* wild type (WT), (**c**) Δ*PKS-NRPS1*, (**d**), Δ*DA*, (**e**) Δ*ER*, (**f**) Δ*TF23* and (**g**) Δ*MFS-T*. The presence of pTET and the correct in locus integration in two independent transformants (T) was checked using primer pairs TET_Seq_F/02222_WT_R (1.50 kb) and Tgluc_hiF/TET_ddr_diag_R (2.10 kb), respectively. The WT or the respective deletion mutant was used as control. M: GeneRuler DNA Ladder Mix.

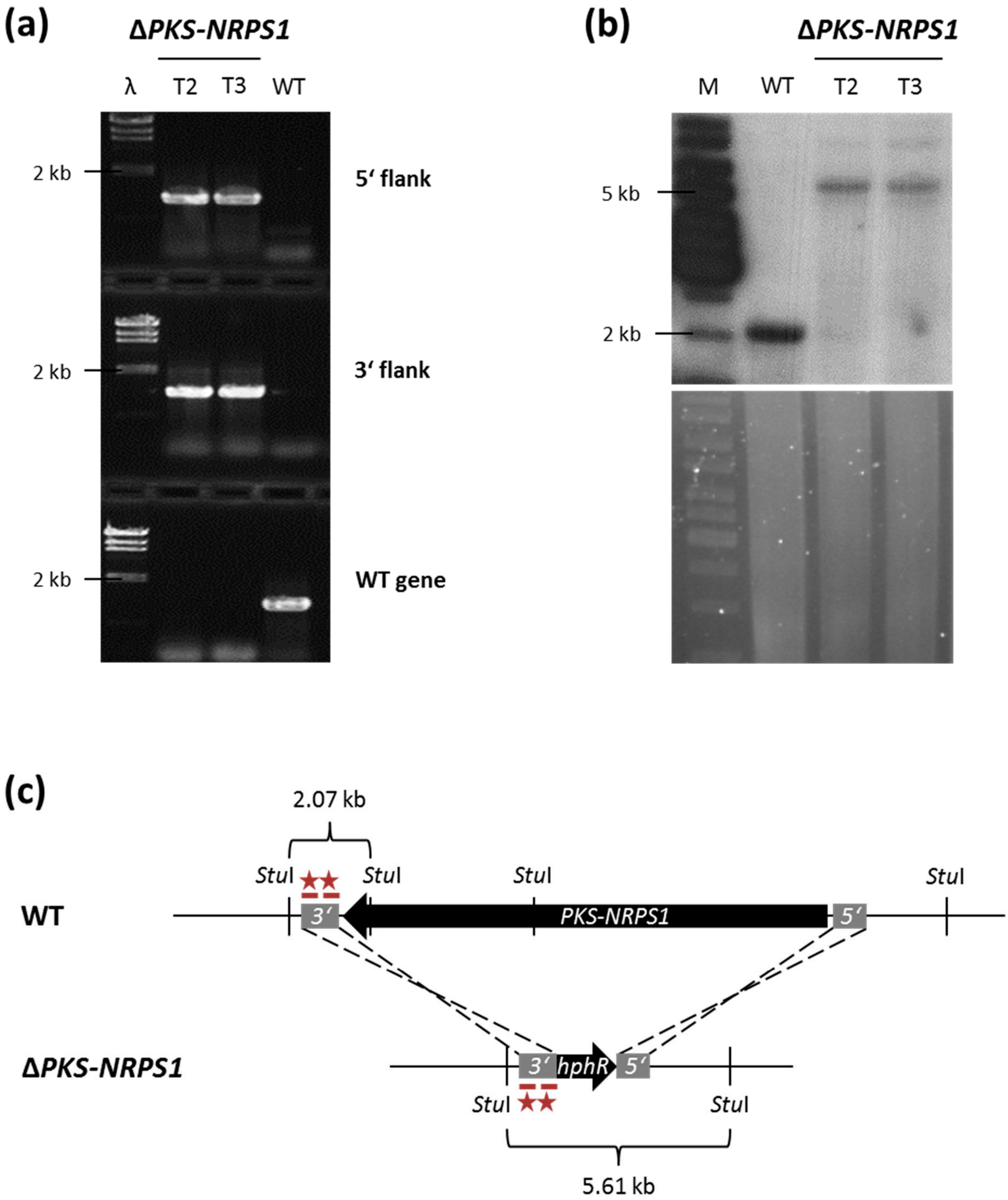

Figure S11. Verification of Δ*PKS-NRPS1* deletion mutants by diagnostic PCR and Southern blot. (**a**) Deletion via homologous recombination with the hygromycin B resistance cassette (*hphR*) was underlined with the amplification of 5′ (trpC_T/02219_5diag) and 3′ (trpC_P2/02219_3diag) flanks but no amplification of wild-type (WT; 02219_WT_F/02219_WT_R) signal for two independent transformants (T). (**b**) For analyzing ectopic integration of deletion constructs, genomic DNA of transformants and WT was digested with *Stu*I and the 3′ flank was applied for probing. (**c**) Detected signals match the expected 2.07 kb for the WT as well as 5.61 kb for Δ*PKS-NRPS1*. λ: λ/*Hin*dIII, M: GeneRuler DNA Ladder Mix.

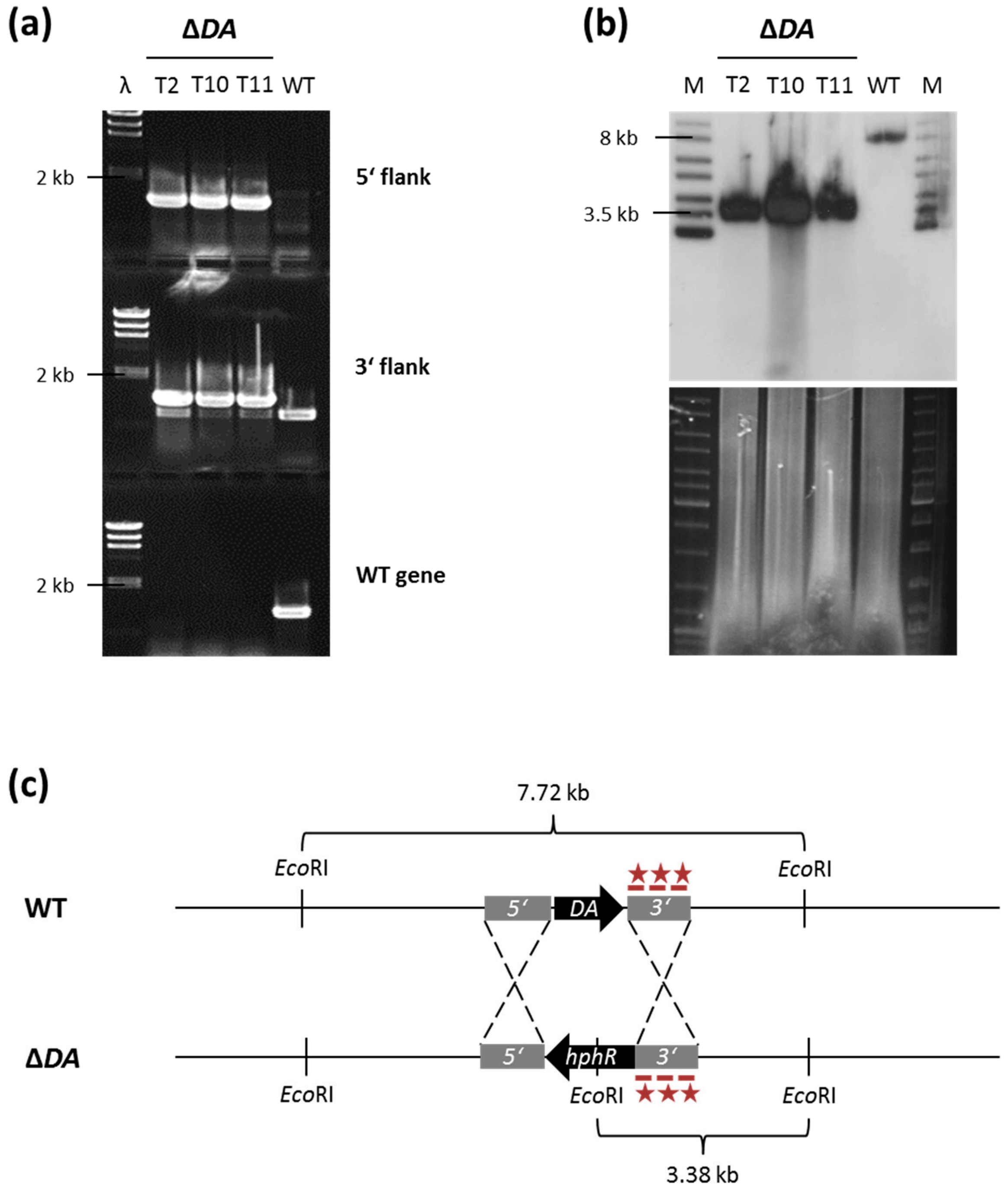

Figure S12. Verification of Δ*DA* deletion mutants by diagnostic PCR and Southern blot. (**a**) Deletion via homologous recombination with the hygromycin B resistance cassette (*hphR*) was underlined with the amplification of 5′ (trpC_T/02220_5diag) and 3′ (trpC_P2/02220_3diag) flanks but no amplification of wild-type (WT; 02220_WT_F/02220_WT_R) signal for three independent transformants (T). (**b**) For analyzing ectopic integration of deletion constructs, genomic DNA of transformants and WT was digested with *Eco*RI and the 3′ flank was applied for probing. (**c**) Detected signals match the expected 7.72 kb for the WT as well as 3.38 kb for Δ*DA*. λ: λ/*Hin*dIII, M: GeneRuler DNA Ladder Mix.

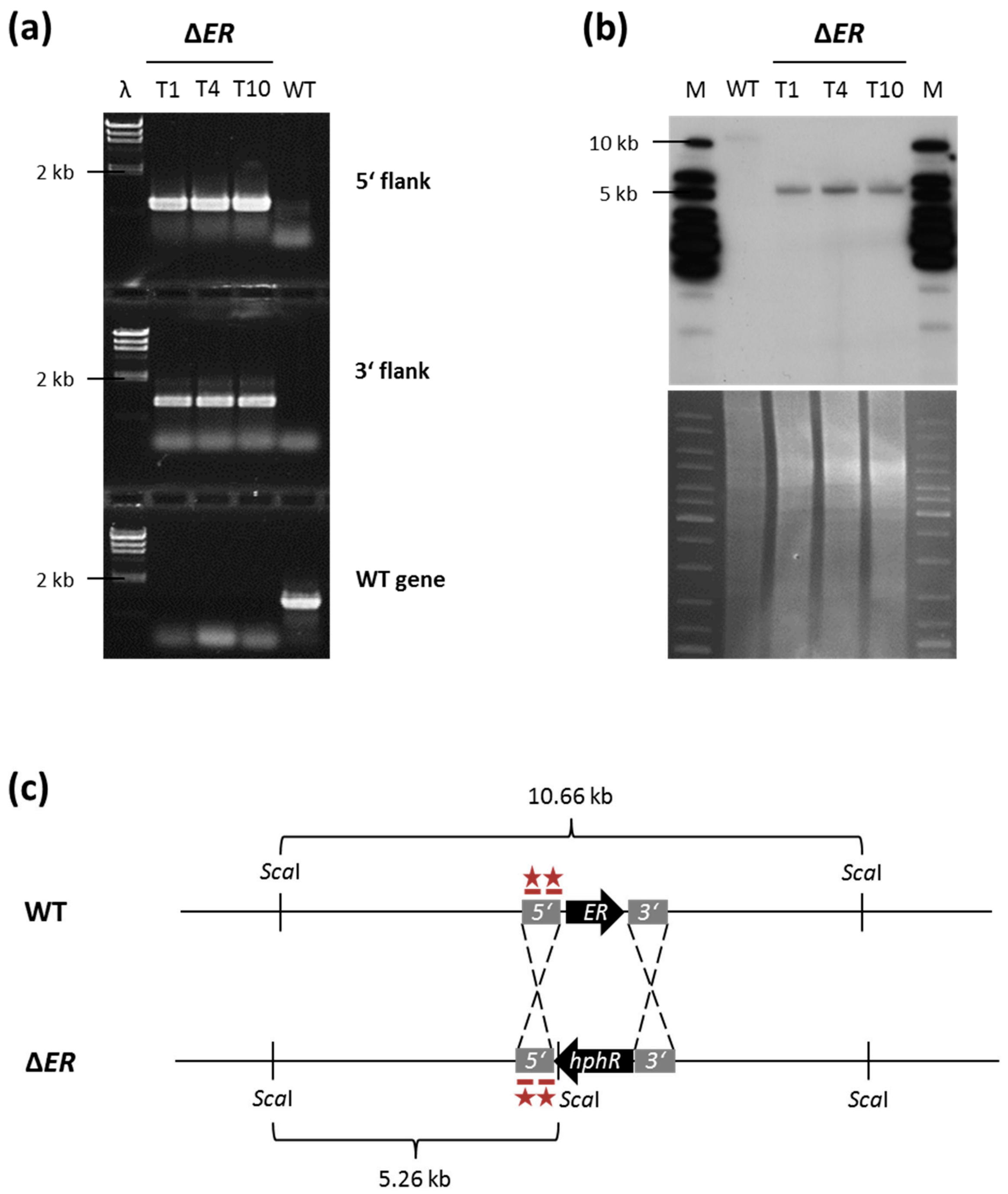

Figure S13. Verification of Δ*ER* deletion mutants by diagnostic PCR and Southern blot. (**a**) Deletion via homologous recombination with the hygromycin B resistance cassette (*hphR*) was underlined with the amplification of 5′ (trpC_T/02221_5diag) and 3′ (trpC_P2/02221_3diag) flanks but no amplification of wild-type (WT; 02221_WT_F/02221_WT_R) signal for three independent transformants (T). (**b**) For analyzing ectopic integration of deletion constructs, genomic DNA of transformants and WT was digested with *Sca*I and the 5′ flank was applied for probing. (**c**) Detected signals match the expected 10.66 kb for the WT as well as 5.26 kb for Δ*ER*. λ: λ/*Hin*dIII, M: GeneRuler DNA Ladder Mix.

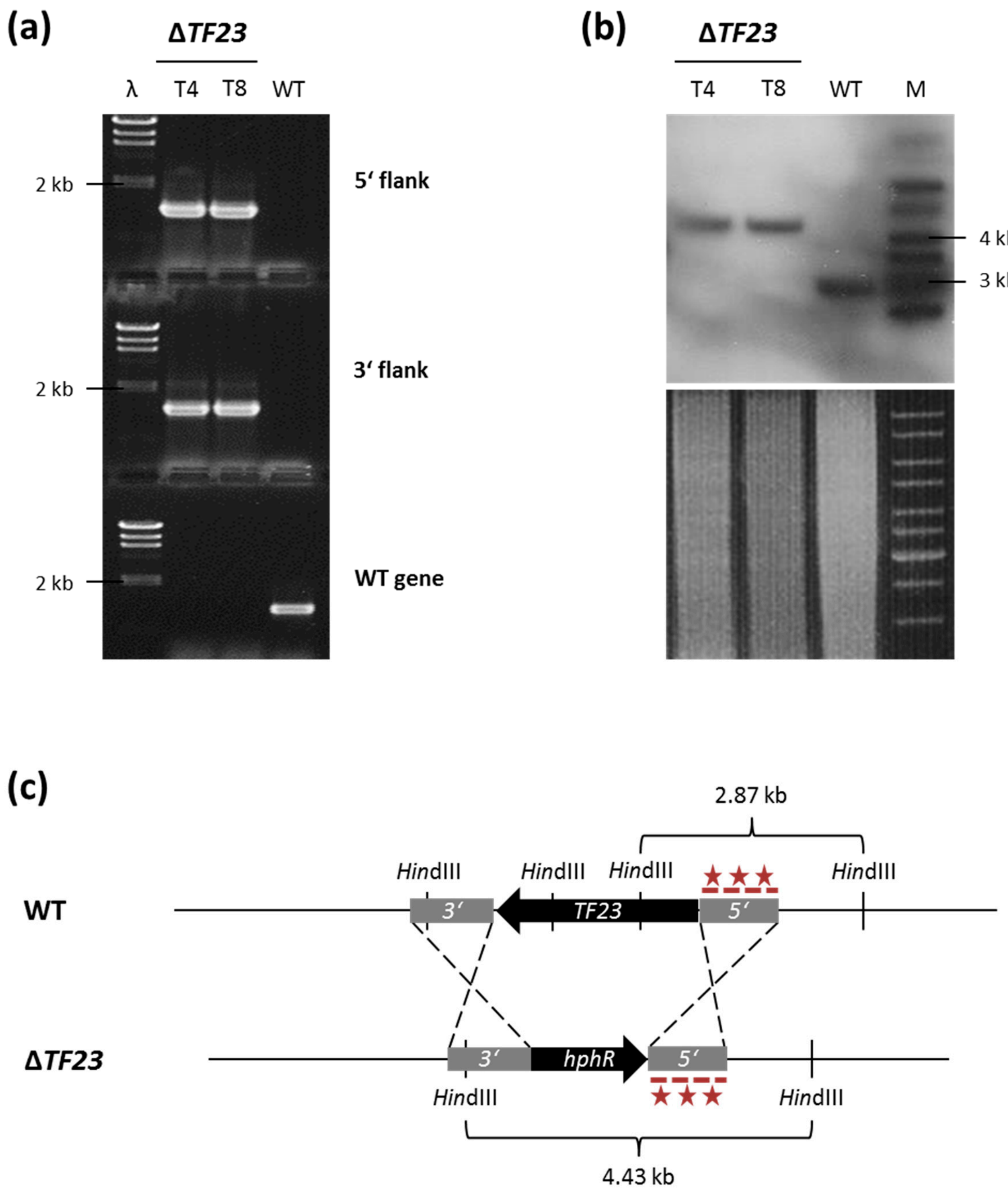

Figure S14. Verification of Δ*TF23* deletion mutants by diagnostic PCR and Southern blot. (**a**) Deletion via homologous recombination with the hygromycin B resistance cassette (*hphR*) was underlined with the amplification of 5′ (trpC_T/02223_5diag) and 3′ (trpC_P2/02223_3diag) flanks but no amplification of wild-type (WT; 02223_WT_F/02223_WT_R) signal for two independent transformants (T). (**b**) For analyzing ectopic integration of deletion constructs, genomic DNA of transformants and WT was digested with *Hin*dIII and the 5′ flank was applied for probing. (**c**) Detected signals match the expected 2.87 kb for the WT as well as 4.43 kb for Δ*TF23*. λ: λ/*Hin*dIII, M: GeneRuler DNA Ladder Mix.

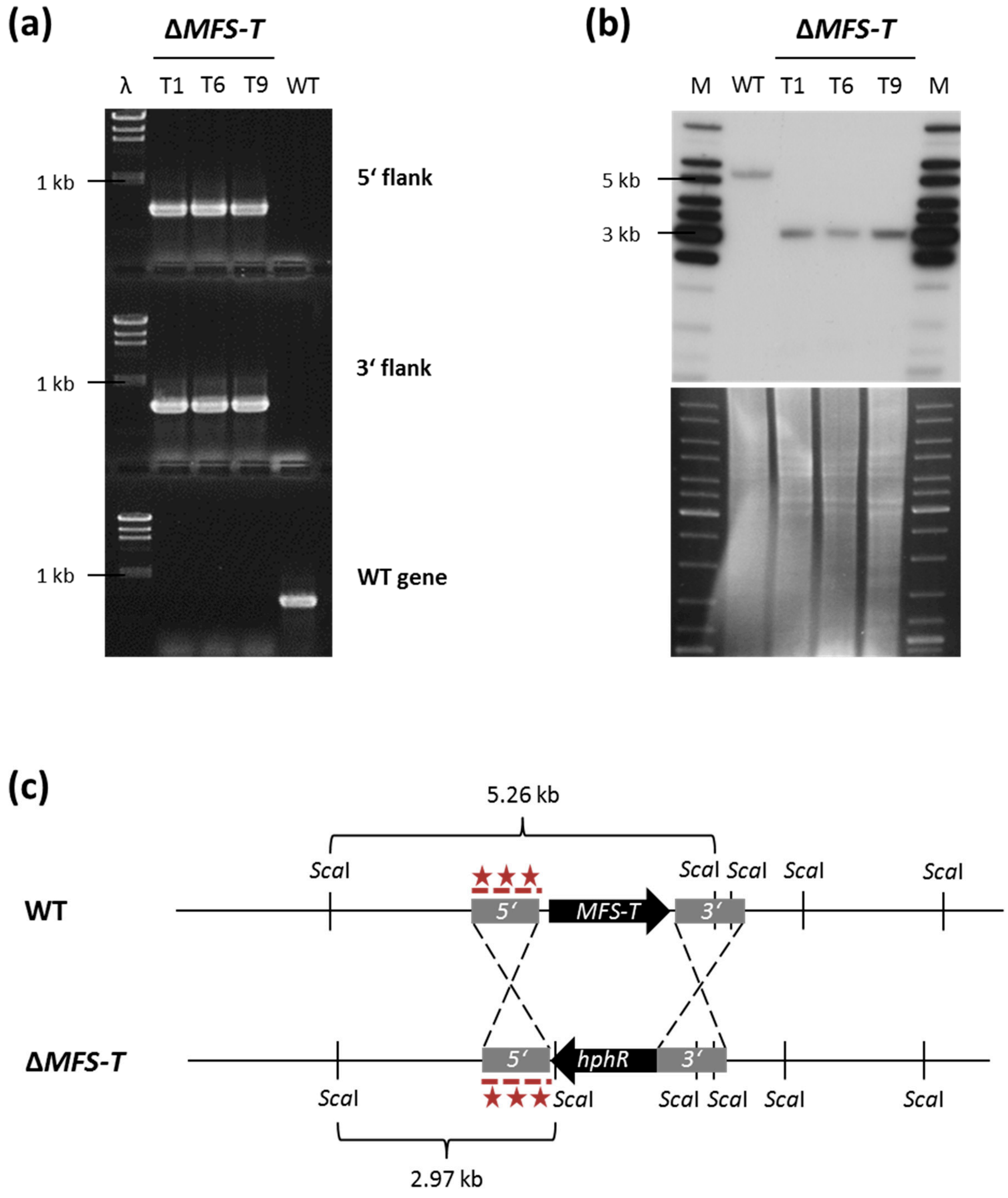

Figure S15. Verification of Δ*MFS-T* deletion mutants by diagnostic PCR and Southern blot. (**a**) Deletion via homologous recombination with the hygromycin B resistance cassette (*hphR*) was underlined with the amplification of 5′ (trpC_T/02224_5diag) and 3′ (trpC_P2/02224_3diag) flanks but no amplification of wild-type (WT; 02224_WT_F/02224_WT_R) signal for three independent transformants (T). (**b**) For analyzing ectopic integration of deletion constructs, genomic DNA of transformants and WT was digested with *Sca*I and the 5′ flank was applied for probing. (**c**) Detected signals match the expected 5.26 kb for the WT as well as 2.97 kb for Δ*MFS-T*. λ: λ/*Hin*dIII, M: GeneRuler DNA Ladder Mix.

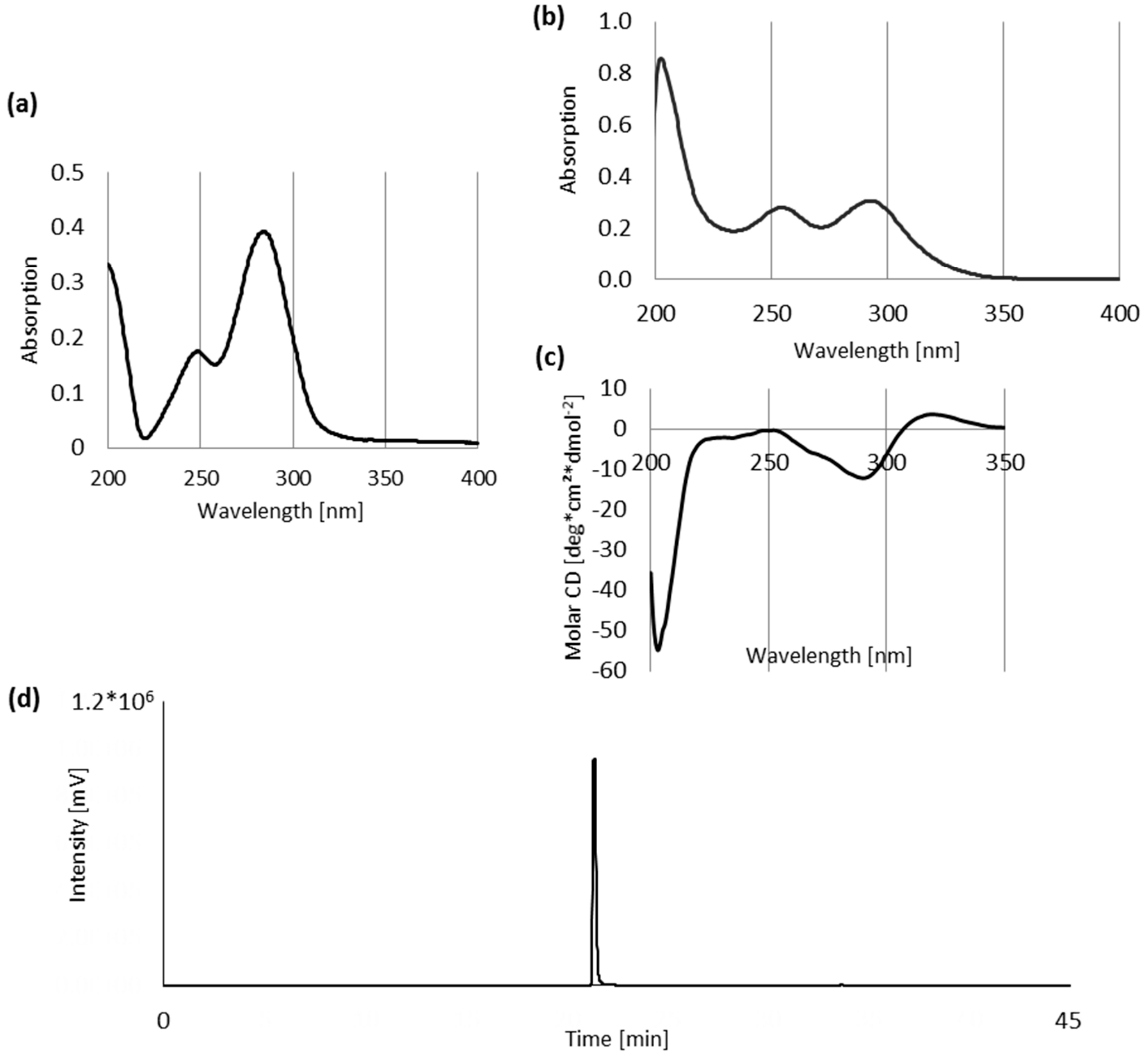

Figure S16. Analysis of physico-chemical properties and purity of trichosetin. (**a**) UV-spectrum of trichosetin in acetonitrile. (**b**) UV-spectrum of trichosetin in methanol. (**c**) Molar CD spectrum of trichosetin in methanol. (**d**) HPLC-ELSD chromatogram of trichosetin, retention time 21.27 min.

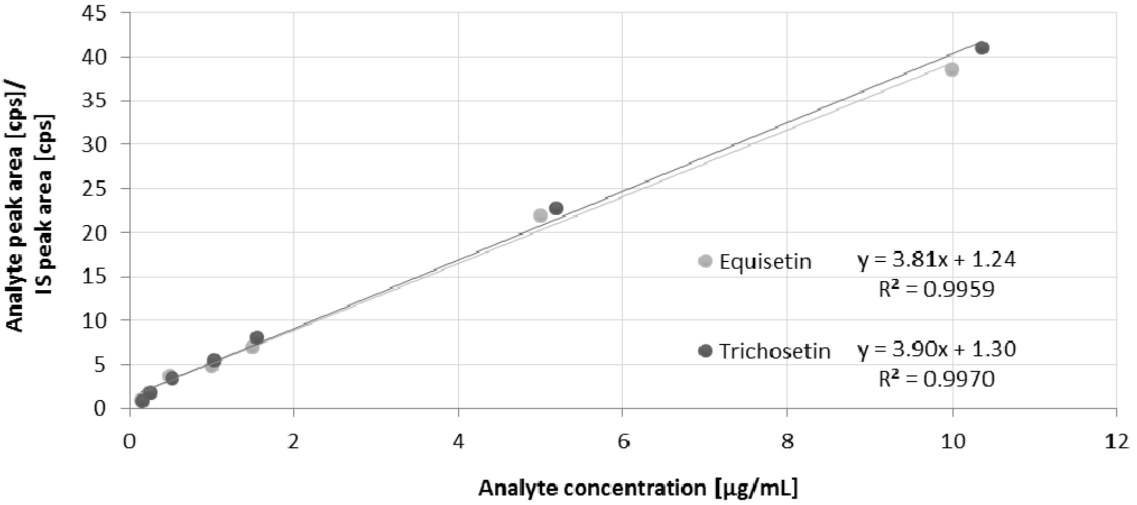

Figure S17. Calibration curve of the semi-quantitative analysis of trichosetin and equisetin, respectively. The analysis was done with HPLC-MS/MS, and OTA was used as internal standard (IS). The corresponding function

of the calibration curves as well as the Pearson correlation coefficient R^2 are given adjacent to the names of the analytes.

Table S1. HPLC-HRMS-CID measurement of trichosetin and the two putative stereoisomers in Δ*DA*/TET::*TF22*. The precursor ion *m/z* 360.22 was fragmented with 40% normalized collision energy (NCE). The highest and second highest fragment ions were fragmented again with 15% NCE. The "?" in the table indicate that the ppm deviation of calculated to measured exact mass was higher than 5 ppm.

**Trichosetin**			**Peak @ 16.43 min**			**Peak @ 18.45 min**		
***m/z* 360.22**	**@CID40**		***m/z* 360.22**	**@CID40**		***m/z* 360.22**	**@CID40**	
***m/z* of product ion**	**Putative loss**	**Intensity [%]**	***m/z* of product ion**	**Putative loss**	**Intensity [%]**	***m/z* of product ion**	**Putative loss**	**Intensity [%]**
342.2062	H_2O	100.0	342.2060	H_2O	100.0	342.2060	H_2O	100.0
332.2217	CO	35.1	332.2216	CO	43.4	332.2216	CO	27.2
175.1478	$C_8H_{11}O_4N$	17.6	175.1478	$C_8H_{11}O_4N$	14.6	175.1478	$C_8H_{11}O_4N$	19.4
205.1948	$C_6H_5O_4N$	9.7	205.1948	$C_6H_5O_4N$	8.8	186.0758	$C_{13}H_{18}$	8.0
186.0758	$C_{13}H_{18}$	6.1	186.0758	$C_{13}H_{18}$	5.7	205.1948	$C_6H_5O_4N$	6.6
231.1741	$C_5H_7O_3N$	5.8	189.1635	$C_{13}H_{18}$	4.8	231.1740	$C_5H_7O_3N$	3.3
189.1635	$C_{13}H_{18}$	4.6	231.1740	$C_5H_7O_3N$	4.4	130.0495	$C_{16}H_{22}O$	2.7
130.0496	$C_{16}H_{22}O$	4.3	130.0495	$C_{16}H_{22}O$	2.8	189.1635	$C_{13}H_{18}$	2.6
***m/z* 332.2**	**@CID15**		***m/z* 332.2**	**@CID15**		***m/z* 332.2**	**@CID15**	
***m/z* of product ion**	**Putative loss**	**Intensity [%]**	***m/z* of product ion**	**Putative loss**	**Intensity [%]**	***m/z* of product ion**	**Putative loss**	**Intensity [%]**
203.1792	$C_5H_7O_3N$	100.0	177.1635	$C_7H_9O_3N$	100.0	177.1635	$C_7H_9O_3N$	100.0
210.1123	C_9H_{14}	47.2	203.1792	$C_5H_7O_3N$	69.2	203.1791	$C_5H_7O_3N$	80.9
177.1635	$C_7H_9O_3N$	41.7	170.0808	$C_{12}H_{18}$	26.4			
191.1790	$C_6H_7O_3N$	23.3	210.1121	C_9H_{14}	20.0			
276.1591	C_4H_8	18.5	191.1793	$C_6H_7O_3N$	16.5			
130.0495	$C_{16}H_{20}$	10.8						
***m/z* 342.2**	**@CID15**		***m/z* 342.2**	**@CID15**		***m/z* 342.2**	**@CID15**	
***m/z* of product ion**	**Putative loss**	**Intensity [%]**	***m/z* of product ion**	**Putative loss**	**Intensity [%]**	***m/z* of product ion**	**Putative loss**	**Intensity [%]**
342.2060	Precursor Ion	100.0	342.2060	Precursor Ion	100.0	342.2061	Precursor Ion	100.0
213.1635	$C_5H_7O_3N$	57.4	213.1636	$C_5H_7O_3N$	61.0	288.1592	C_4H_6	70.0
286.1435	C_4H_8	46.4	300.1591	C_3H_6	50.6	300.1591	C_3H_6	69.5
288.1591	C_4H_6	45.0	288.1591	C_4H_6	48.3	213.1634	$C_5H_7O_3N$	59.1
300.1591	C_3H_6	42.7	286.1433	C_4H_8	47.7	286.1434	C_4H_8	53.8
187.1478	$C_7H_9O_3N$	30.4	324.1954	?H_2O	45.8	324.1955	?H_2O	37.8
324.1959	?H_2O	29.8	187.1477	$C_7H_9O_3N$	45.5	187.1478	$C_7H_9O_3N$	28.9
312.1956	CH_2O	29.5	312.1953	CH_2O	39.8	272.1278	C_5H_{10}	25.1
173.1321	$C_8H_{11}O_3N$	28.3	173.1320	$C_8H_{11}O_3N$	33.6	173.1323	$C_8H_{11}O_3N$	23.0
201.1635	$C_6H_7O_3N$	25.9	201.1635	$C_6H_7O_3N$	27.7	201.1636	$C_6H_7O_3N$	23.0
272.1278	C_5H_{10}	24.8	170.0807	$C_{13}H_{16}$	20.7	312.1957	CH_2O	22.8
246.1123	C_7H_{12}	19.1	156.0650	$C_{14}H_{18}$	13.7	274.1437	C_5H_8	17.9
274.1436	C_5H_8	15.1	246.1123	C_7H_{12}	11.6	246.1120	C_7H_{12}	10.4
170.0808	$C_{13}H_{16}$	13.4	272.1279	C_5H_{10}	9.7	170.0812	$C_{13}H_{16}$	7.0
234.1124	C_8H_{12}	12.7				234.1124	C_8H_{12}	6.0
159.1165	$C_9H_{13}O_3N$	12.6						
156.0652	$C_{14}H_{18}$	12.2						

Table S2. Primer sequences used for the generation of deletion constructs, verification of their homologous integration as well as for probe generation. Introduced overhangs required for yeast recombinational cloning are underlined.

Gene	Primer	Sequence 5' → 3'
02218	02218_WT_F	GTTAGGCATCAAGTCCATTCTCC
	02218_WT_R	GCGAGAGATTCGTTAAAGCGC
PKS-NRPS1 (*02219*)	02219_5F	GTAACGCCAGGGTTTTCCCAGTCACGACGCCAGATGCATGGTACCATC
	02219_5R	ATCCACTTAACGTTACTGAAATCTCCAACGATTGATCGAGCAGTTGACC
	02219_3F	CTCCTTCAATATCATCTTCTGTCTCCGACCAAGTTCCTAAGAGGCCG
	02219_3R	GCGGATAACAATTTCACACAGGAAACAGCGGCTACGTAATGCAGCTTG
	02219_5diag	GCGCGAGGACCTAGCTCAGG
	02219_3diag	TCGGCATGTTGGTTAACGGC
	02219_WT_F	CCGAGACACACAAGGGACAGCC
	02219_WT_R	CCTGGAAGGCATCGAGCTCAC
DA (*02220*)	02220_5F	GTAACGCCAGGGTTTTCCCAGTCACGACGCCAAGCTTGATGAACAGGCCG
	02220_5R	ATCCACTTAACGTTACTGAAATCTCCAACGAGTCAAGAGTAATTGGGTCG
	02220_3F	CTCCTTCAATATCATCTTCTGTCTCCGACTCATTTGTTAGTAACTGGTGG
	02220_3R	GCGGATAACAATTTCACACAGGAAACAGCCGGGATTTACCGAAACAGC
	02220_5diag	GTTGGCTCCAGCTGCGATGG
	02220_3diag	CGACTGCACCGGGTGTGACG
	02220_WT_F	GGCTCAGGCAATGTCTTCGCC
	02220_WT_R	CGCCTCCTCATCCGCACC
ER (*02221*)	02221_5F	GTAACGCCAGGGTTTTCCCAGTCACGACGGATTTAGGTGCCGAGGTCTTG
	02221_5R	ATCCACTTAACGTTACTGAAATCTCCAACCTTGAGTTGACAAGAACGC
	02221_3F	CTCCTTCAATATCATCTTCTGTCTCCGACCAGTCTTCCCAACATCATAAGC
	02221_3R	GCGGATAACAATTTCACACAGGAAACAGCCTAGGATGCATACTACAGACTC
	02221_5diag	GGTGAAACTGACAGGGTTGAATG
	02221_3diag	CAACCAAGGTTAGGTCGCTC
	02221_WT_F	CGCCTTGGTGGGCACTCC
	02221_WT_R	GACCTCTGCAAGACCACCCTGC
TF22 (*02222*)	02222_WT_F	GCACACTCCGCCACATGCC
	02222_WT_R	GCCTTGAGCGACCTAACCTTGG
TF23 (*02223*)	02223_5F	GTAACGCCAGGGTTTTCCCAGTCACGACGGATAGATGATGAGACGCCC
	02223_5R	ATCCACTTAACGTTACTGAAATCTCCAACGACGCGATTCCTGGTCCGCC
	02223_3F	CTCCTTCAATATCATCTTCTGTCTCCGACGATTGTTTCGGCTACAAAGG
	02223_3R	GCGGATAACAATTTCACACAGGAAACAGCCGGTACACAATCAACCAACCG
	02223_5diag	GCACAGCCGATTGTGAAGGCC
	02223_3diag	CGTGAGGAGTCAGTTACGACGG
	02223_WT_F	GCTGTTCTCGACGGGATTGCC
	02223_WT_R	CGGCGTCAGTTCTGTTTCTGGC
MFS-T (*02224*)	02224_5F	GTAACGCCAGGGTTTTCCCAGTCACGACGGTAGCAACAGCCGTGTTCACG
	02224_5R	ATCCACTTAACGTTACTGAAATCTCCAACCTGGGATGATAACACTGC

	02224_3F	CTCCTTCAATATCATCTTCTGTCTCCGACGTACATACTGGGCTTGACAAG
	02224_3R	GCGGATAACAATTTCACACAGGAAACAGCCATATCCGAGGATACAGGGATC
	02224_5diag	CCTCCGCCCGTGGATCG
	02224_3diag	CTAGCGGCCATATTTCGGC
	02224_WT_F	CCTGGGCTTCGCGCTAGG
	02224_WT_R	GTGCCGATGATAGGGACGATCC
02225	02225_WT_F	CGGCGGCCTAGCTGCCC
	02225_WT_R	CGCTAGGAGACTGAGCGAGTTGC
hphR	hph_F	GTCGGAGACAGAAGATGATATTGAAGGAGC
	hph_R	GTTGGAGATTTCAGTAACGTTAAGTGGAT
	trpC_T	GGAATAGAGTAGATGCCGACCGG
	trpC_P2	GTGATCCGCCTGGACGACTAAACC

Table S3. Primer sequences used for the generation and analysis of constitutive and inducible overexpression vectors. Introduced overhangs required for yeast recombinational cloning are underlined.

Gene	Primer	Sequence 5' → 3'
pOE::*TF22*	TF22_OE_F	CCATCACATCACAATCGATCCAACCATGTCCACACGGAACAGC
	TF22_OE_R	GTAACGCCAGGGTTTTCCCAGTCACGACGGGCTCCTGGTTGACGGACGC
	TF22_OE_diag	GGACCGGTCGTGGTCGCC
	TF22_Seq	GCACACTCCGCCACATGCC
pOE::*TF23*	TF23_OE_F	CCATCACATCACAATCGATCCAACCATGGAGTGGGGTCCAGGG
	TF23_OE_R	GTAACGCCAGGGTTTTCCCAGTCACGACGGCGTCTCCAGCACGTAAGGG
	TF23_Seq	GCTGTTCTCGACGGGATTGCC
	TF23_OE_diag	CGGCGTCAGTTCTGTTTCTGGC
pOE::*eqxD*	eqxD_OE_F	CCCCGTATCACAACCACATTCACAATGTCATCTATCCTTTCGCG
	eqxD_OE_R	GTTGACATGGAGCTATTAAATCATCAACTCTGTACAGGTAGC
	eqxD_Seq1	GCCACCAAGACGGGCATGG
	eqxD_Seq2	CTTCGGCATGTGTGCTGCAGG
PoliC	PoliC_Seq_F2	GGGAGACGTATTTAGGTGCTAGGG
PglnA	GS_Prom_M	ATGTCGAAGTATCTTCCCTGTGC
pTET::*TF22*	TET-A-PoliC-F	CATCACATCACAATCGATCCAACCATGTCTAGACTGGACAAGAGCAAAGT
	TET-A-R	GCCTCGTGATACGCACGGCCGCATGATTC
	TET-B-F	GAATCATGCGGCCGTGCGTATCACGAGGC
	TET-B-GFP-R	TTACTTACCTCACCCTTGGAAACCATGGAAACGGTGATGTCTGCTCAAGC
	TET_ddr_F	GATTTGACAACCCCTTCCCCCCAACAAGATTTAGTAGTCGTTGTCACCAC
	TET_ddr_R	GTAACGCCAGGGTTTTCCCAGTCACGACGCTTGATCTGAGTCGATCACC
	TF22_TET_F	CCCGCTTGAGCAGACATCACCGTTTATGTCCACACGGAACAGCC
	TF22_TET_R	TAATCATACATCTTATCTACATACGTCAAAGATTCATCTTTTCTC
	TET_Seq_F	ATTCATCTTCCCATCCAAGAACC
	Tgluc_hiF	CATACGTACATCTGATTTGACAACC
	TET_ddr_diag_R	CTTTCATGGCGAAGCTTCAGGC

Table S4. Primer sequences used for expressional analysis by quantitative real-time PCR. Reference genes: *GMT*, GDP mannose transporter gene; *RAC*, related actin gene; *UBI*, ubiquitin gene.

Gene	Primer	Sequence 5' → 3'
PKS-NRPS1 (*02219*)	PKS-NRPS1_RT_F	CTGCTGGTTCAATCGGCCTTCC
	PKS-NRPS1_RT_R	CGGACGCCAAGGAACTTGACG
DA (*02220*)	02220_RT_F	CGCTACGACGCATCCTCTGAGG
	02220_RT_R	GCCCAGGCGCACTCGTAGG
ER (*02221*)	02221_RT_F	GTCAAGTCCTACGGCGCCAGC
	02221_RT_R	CGCCACTGCCTCAGAGTATGGC
TF22 (*02222*)	02222_RT_F	GCTTGCAGCTCGGAGAACTGCC
	02222_RT_R	GCTCAGCGCTGAAGTCCATCCC
TF23 (*02223*)	02223_RT_F	CCGGTCTCGGCTCACAGTTTCC
	02223_RT_R	GCAGGTTCCATGGCCATGCC
MFS-T (*02224*)	02224_RT_F	GCTGGCACGTGCCATTGTACG
	02224_RT_R	GGAGCGGCATTCTCTTCGCC
eqxD	eqxD_RT_F	GGCTCATCTGGAAGGAGGTTCTCG
	eqxD_RT_R	CGCAAGGTGCAGAAAGTCGGTTC
GMT (*07710*)	FGMTRTPCRFW	CGGGCCATTCTCTATTCTTTC
	FGMTRTPCRRV	ATGCTGTGATGGCAACAATG
RAC (*05652*)	FRACRTPCRFW	GAGAACGAGCGTGTCTTGATTGAGCC
	FRACRTPCRRV	TTTCCTCCGCAGAATGAAGAAGGACTC
UBI (*08398*)	FUBRTPCRFW	CCAACCCTGACGATCCTCTTGTGC
	FUBRTPCRRV	TACTTTCGAGTCCACTCCCGAGCTG

Table S5. NMR spectra of trichosetin in MeOH-d_4, measured with a 600 MHz NMR-spectrometer and referenced to tetramethylsilane. Signals are given in ppm. The number of the corresponding carbon atoms (no. of C) is similar to that reported by Marfori et al. [28]. Multiple proton signals are divided by a semicolon.

no. of C	$\delta^{13}C$	δ^1H, multiplicity, J (Hz)	gHMBC	gCOSY	NOESY
1	204.1, C	-	-	-	-
2	51.4, C	-	-	-	-
3	43.6, CH	3.38, m	-	CH-4, CH-13/14	CH-13/14, CH-4, CH_3-12
4	131.7, CH	5.54, m	C-3, C-6, C-2	CH-3, CH-5	CH-3, CH-5
5	130.4, CH	5.32, m	C-6, C-11, C-7, C-3	CH-4	CH_2-7, CH-4
6	36.1, CH	2.12, broad s	-	CH_2-7	CH_2-7 (weak)
7	41.7, CH_2	1.11, td, 12.72, 12.65, 5.09; 1.58, m	C-16, C-8, C-6, C-11, C-5	CH-6, CH-8 (weak)	1.58 ppm: CH_3-16
8	29.6, CH	1.35, m	overlapped by singulett CH_3-12	CH_3-16	CH_3-16
9	36.5, CH_2	1.67**, d; 0.88, qd, 23.68, 12.55, 12.55, 3.19	0.88 ppm: C-10, C-8, C-11	0.88 ppm: CH_2-10	Problems with overlapping signals

10	24.0, CH_2	1.63, m; 1.47, m	C-9, C-11	CH_2-9	1.63 ppm: CH-13/14, CH_3-12
11	38.5, CH	2.68, broad s	-	-	-
12	18.9, CH_3	1.32, s	C-11, C-3, C-2, C-1	-	CH_2-10, CH-13/14, CH-3
13	128.3, CH	5.46*, m	C-15, C-3, C-4, C-14	CH_3-15, CH-3	CH_3-12, CH_2-10, CH_3-15, CH-3
14	133.3, CH	5.46*, m	C-15, C-3, C-4, C-13		
15	18.2, CH_3	1.67**, d, 4.36	C-13, C-14	CH-13/14	
16	23.0, CH_3	0.82, d, 6.5	C-8, C-9, C-7	CH-8	CH-8
2'	nd	-	-	-	
3'	nd	-	-	-	
4'	192.3, C	-	-	-	
5'	64.9, nd	nd	-	-	
6'	62.4, CH_2	3.77, dd, 11.53, 4.86; 3.84, dd, 11.51, 2.91	C-4', C-5'		

* = cannot be separated
** = signals overlapping
nd = not detected

Table S6. NMR spectra of trichosetin in MeOH-d4, measured with a 600 MHz NMR-spectrometer and referenced to tetramethylsilane, in comparison to NMR spectra found in the literature. Signals are given in ppm. The number of the corresponding carbon atoms (no. of C) is similar to that reported by Marfori et al. [28]. Multiple proton signals are divided by a semicolon.

	NMR data obtained in this study		Kakule et al., 2013		Marfori et al., 2002	
**no. of C**	**$\delta^{13}C$**	**$\delta^{1}H$, multiplicity, J (Hz)**	**$\delta^{13}C$**	**$\delta^{1}H$, multiplicity, J (Hz)**	**$\delta^{13}C$**	**$\delta^{1}H$**
1	204.1, C	-	204.6		201.5	
2	51.4, C	-	51.5		50	
3	43.6, CH	3.38, m	46.2	3.46, br	46.2	3.43
4	131.7, CH	5.54, m	133.4	5.15, m	132.3	5.23
5	130.4, CH	5.32, m	132.6	5.41, m	131.2	5.44
6	36.1, CH	2.12, broad s	39.9	1.86, m	39.8	1.86
7	41.7, CH_2	1.11, td, 12.72, 12.65, 5.09; 1.58, m	43.6	1.89, m; 0.89, m	43.4	1.83; 0.86
8	29.6, CH	1.35, m	34.8	1.53, m	34.8	1.49
9	36.5, CH_2	1.67**, d; 0.88, qd, 23.68, 12.55, 12.55, 3.19	36.9	1.77, m; 1.11 m	36.9	1.78; 1.10
10	24.0, CH_2	1.63, m; 1.47, m	29.5	2.01, br; 1.08, br	29.2	2.02; 1.07
11	38.5, CH	2.68, broad s	41.3	1.68, m	41.0	1.64
12	18.9, CH_3	1.32, s	14.2	1.45, s	13.7	1.42
13	128.3, CH	5.46*, m	128.0	5.37, m	127.6	5.38
14	133.3, CH	5.46*, m	127.9	5.26, m	127.9	5.14
15	18.2, CH_3	1.67**, d, 4.36	17.8	1.52, d, 5.7	18.6	1.56

16	23.0, CH_3	0.82, d, 6.5	23.0	0.94, d, 6.8	23.2	0.94
2'	nd	-	nd		180.9***	
3'	nd	-	nd		100.7***	
4'	192.3, C	-	nd		192.9***	
5'	64.9, nd	nd	nd	nd	64.5	3.76
6'	62.4, CH_2	3.77, dd, 11.53, 4.86; 3.84, dd 11.51, 2.91	62.2	3.79, m; 3.74, m	61.9	3.82; 3.79

* = cannot be separated
** = signals overlapping
*** = detected at -80 °C
nd = not detected
